# RNA G-quadruplex structures exist and function in vivo in plants

**DOI:** 10.1186/s13059-020-02142-9

**Published:** 2020-09-01

**Authors:** Xiaofei Yang, Jitender Cheema, Yueying Zhang, Hongjing Deng, Susan Duncan, Mubarak Ishaq Umar, Jieyu Zhao, Qi Liu, Xiaofeng Cao, Chun Kit Kwok, Yiliang Ding

**Affiliations:** 1 Department of Cell and Developmental Biology, John Innes Centre, Norwich Research Park, Norwich, NR4 7UH UK; 2 College of Life Sciences, University of Chinese Academy of Sciences, Beijing, 100049 China; 3 State Key Laboratory of Plant Genomics, Institute of Genetics and Developmental Biology, CAS Center for Excellence in Molecular Plant Sciences, Chinese Academy of Sciences, Beijing, 100101 China; 4 Department of Chemistry, City University of Hong Kong, Kowloon Tong, Hong Kong SAR, China; 5 Present Address: School of Life Sciences, University of Sussex, Brighton, BN1 9QG UK; 6 Shenzhen Research Institute of City University of Hong Kong, Shenzhen, 518057 China

**Keywords:** RNA G-quadruplex structure, rG4-seq, Selective 2′-hydroxyl acylation with lithium ion-based primer extension (SHALiPE), Gene regulation, Plant development

## Abstract

**Background:**

Guanine-rich sequences are able to form complex RNA structures termed RNA G-quadruplexes in vitro. Because of their high stability, RNA G-quadruplexes are proposed to exist in vivo and are suggested to be associated with important biological relevance. However, there is a lack of direct evidence for RNA G-quadruplex formation in living eukaryotic cells. Therefore, it is unclear whether any purported functions are associated with the specific sequence content or the formation of an RNA G-quadruplex structure.

**Results:**

Using rG4-seq, we profile the landscape of those guanine-rich regions with the in vitro folding potential in the *Arabidopsis* transcriptome. We find a global enrichment of RNA G-quadruplexes with two G-quartets whereby the folding potential is strongly influenced by RNA secondary structures. Using in vitro and in vivo RNA chemical structure profiling, we determine that hundreds of RNA G-quadruplex structures are strongly folded in both *Arabidopsis* and rice, providing direct evidence of RNA G-quadruplex formation in living eukaryotic cells. Subsequent genetic and biochemical analyses show that RNA G-quadruplex folding is able to regulate translation and modulate plant growth.

**Conclusions:**

Our study reveals the existence of RNA G-quadruplex in vivo and indicates that RNA G-quadruplex structures act as important regulators of plant development and growth.

## Background

The in vivo folding of RNA structure is tightly associated with its function and largely dependent on the cellular context [1, 2]. Because of the complexity within living cells, RNA folding in vivo could be very different from that in vitro [3, 4]. For many complex RNA structures, although their folding in vitro has been widely studied, the folding in vivo is poorly understood. One of such complex structures is RNA G-quadruplex (RG4), which is folded with guanine-rich (G-rich) sequences in vitro and consists of two or more layers of G-quartets involving both Hoogsteen and Watson-Crick base pairs [5, 6]. RG4s can be very stable in vitro in the presence of cations such as potassium (K^+^); therefore, they are hypothesized to exist in vivo and to be involved in novel functions [5], such as post-transcriptional regulation of gene expression [7–9]. Nevertheless, the lack of direct evidences of RG4 folding in vivo raised the key question of whether all these suggested functions are due to RG4 or sequence motif. For example, a (GGC)_4_ motif was proposed to fold into an RG4 to repress the translation of tumour-related genes [8]. However, without the evidence of in vivo folding, the translation inhibition may simply be due to the (GGC)_4_ sequence motif. Also, emerging evidences argue that this sequence motif is likely to form a stable hairpin RNA secondary structure rather than RG4 [10]. Hence, it is crucial to determine whether RG4 truly exists in vivo, such that one can investigate and establish the relationship between RG4 and associated biological functions.

In recent decades, numerous efforts have been made to detect the folding of RG4s in fixed or living cells using G-quadruplex-specific antibodies [11], ligands [12–14] and fluorescent probes [15, 16]. However, these methods suffer from three major shortcomings. Firstly, the antibodies/ligands/probes may induce the structure formation by perturbing the RNA G-quadruplex folding equilibrium in cells or binding to the G-rich sequence motifs [17, 18]. Secondly, these methods cannot quantitatively determine the folding state of individual G-rich regions of interest in cells. Thirdly, these methods cannot exclude the possibility of RG4 folding in fixing, permeabilizing or staining cells [17]. Because of these considerable shortcomings, these methods are considered inadequate for robustly determining the existence of RG4s and actual folding state of G-rich regions in living cells.

To address these shortcomings, two methods based on RNA chemical structure probing have been developed to determine the folding state of G-rich regions in living cells. One direct method is based on the property of the chemical 2-methylnicotinic acid imidazolide (NAI) which preferentially modifies the last G residue of each G-tracts, which may be due to the exposure of the 2′-hydroxyl of last G residues when RG4 is folded [17, 19]. This special modification pattern is subsequently detectable by reverse transcription at both individual targeted RNAs and at a genome-wide scale [17, 19]. The other method is more indirect; the modification is based on the specific methylation of the N7 position of guanine (N7G) by dimethyl sulfate (DMS) [20]. When a very high concentration of DMS (~ 8%) is applied to the cells, all the N7 positions of G residues in the unfolded G-rich regions will be methylated in vivo [17]. These methylated G-rich regions are unable to refold into RG4 in vitro in the presence of K^+^. Since RG4 refolding in vitro is able to stall reverse transcriptase during reverse transcription (RT) [21], in vivo unfolded G-rich regions are unable to lead to RT stalling [17]. On the opposite, if the G-rich regions are folded into RG4s in vivo, then N7G is protected from DMS modification. These unmodified G-rich regions are able to reform into RG4s later during RT, subsequently causing the RT stalling [17]. Both methods were applied in yeasts, and the DMS method was also applied in mouse embryonic stem cells (mESCs) [17]. These studies concluded that G-rich regions with the potential to form RG4s in vitro were globally unfolded in vivo. These results underpinned the fact that both yeast and mice avoid the formation of RG4s in vivo and the presumption that RG4s do not exist in eukaryotic cells [17].

Unlike yeasts and animals, plants are sessile eukaryotic organisms and have evolved independently with unique regulatory strategies. For instance, cellular K^+^ is the most abundant ion in plants and plays key roles in plant development and stress response [22, 23]. Given the importance of K^+^ in affecting RG4 folding, and the ability of plants in maintaining K^+^ balance within the cells, we hypothesize that plants are more likely to adopt RG4 in vivo. In addition, our previous study and others on individual G-rich sequences with folding potential in vitro have suggested that plants might favour RG4s [9, 24]. However, the existence of RG4s in vivo in plants has not been determined and remains an open question.

Here, we investigated the in vivo folding state of G-rich regions in plants. Firstly, we profiled the landscape of the G-rich regions with the potential to fold into RG4s in vitro in the *Arabidopsis* transcriptome. We also revealed the unique RNA structural features of these regions. Using chemical structure probing, we found that those G-rich regions with in vitro folding potential are strongly folded in both *Arabidopsis* and rice. We further demonstrated that RG4 formation is sufficient to regulate gene expression and subsequently plant growth. Taken together, these findings provided the first direct evidence of global RG4 formation in living eukaryotic cells and revealed RG4s as important regulators for plant growth.

## Results

### Profiling of G-rich regions with potential to fold into RG4s in *Arabidopsis* transcriptome

**Fig. 1:**
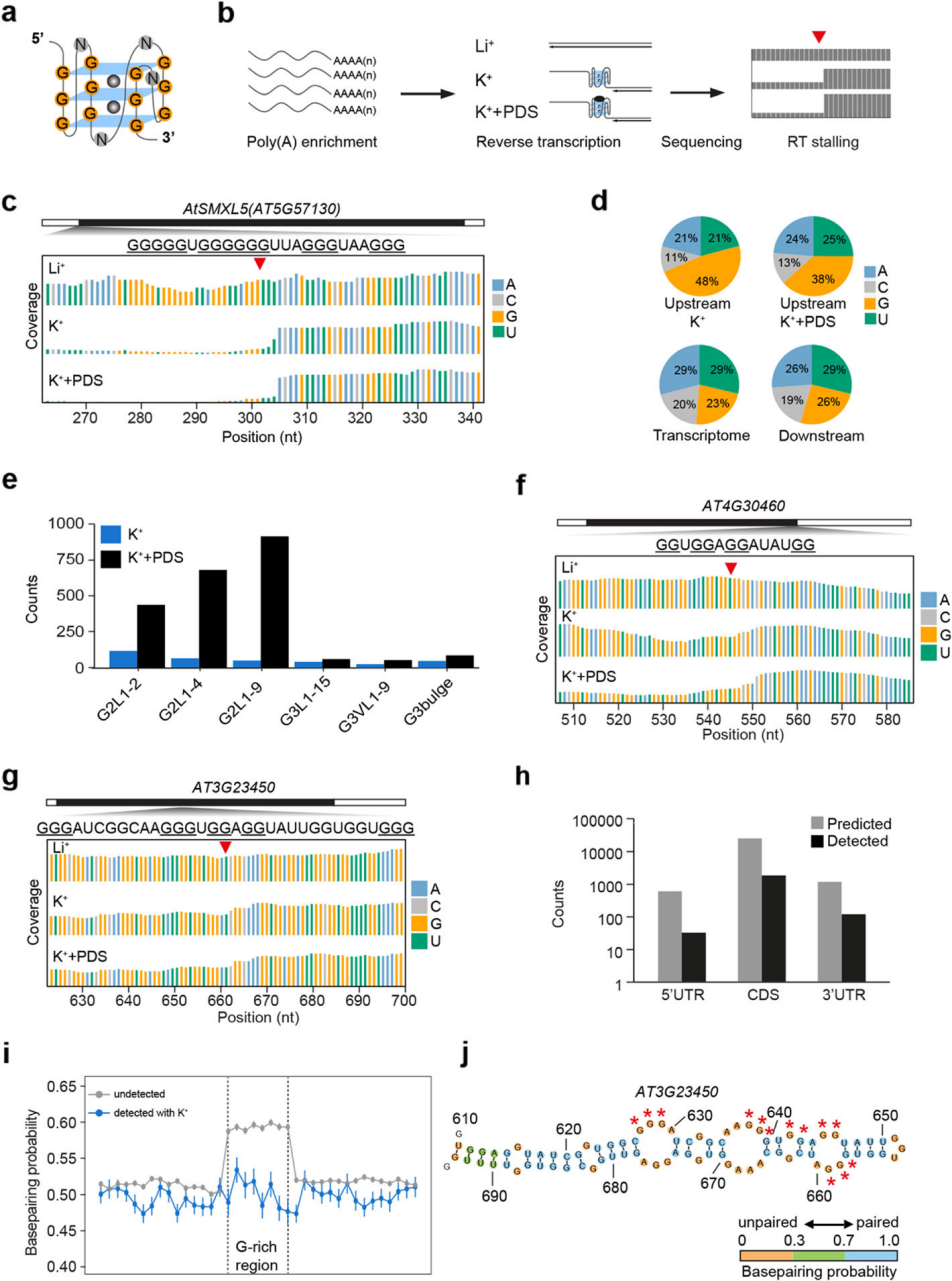
rG4-seq reveals the global landscape of G-rich regions with the potential to fold into RG4s in *Arabidopsis*. **a** Schematic of an RNA G-quadruplex (RG4). The schematic depicts an RG4 with three layers of G-quartets (G3 RG4, guanine (G) coloured in orange), with the loop length of any nucleotide (N, coloured in grey), potassium ions (K^+^, grey ball) coordinated within the G-quartets stabilize RG4s. **b** Workflow of rG4-seq. Poly(A)-enriched RNAs were subjected to reverse transcription under the buffers with Li^+^ (non-stabilizing condition), K^+^ (stabilizing condition) or K^+^+PDS (stronger stabilizing condition), respectively. The G-rich region sites with folding potential were identified by comparing the coverage of reads between the rG4-seq libraries with different cations as described above. **c** rG4-seq profiles of *AtSMXL5* displayed the read coverage of reverse transcription (RT) with Li^+^ (top), K^+^ (middle) and K^+^+PDS (bottom), respectively. The 3′end of the G-rich region is indicated by a red triangle. A (blue), C (light grey), G (yellow) and U (green). **d** Residue distribution around RTS sites. Guanine (G) was strongly enriched in the upstream sequences of RT stalling (RTS) identified under both K^+^ and K^+^+PDS conditions, but not in the transcriptome and the downstream sequences of RTS. A (blue), C (light grey), G (yellow) and U (green). **e** Classification of G-rich regions with folding potential. G-rich regions with folding potential identified in K^+^ (dark blue) and K^+^+PDS conditions (black) were classified into six categories according to the number of G-quartets (G2 with two G-quartets or G3 with three G-quartets), loop length (L, 1–15 nt) and bulge size (non-canonical G3 RG4s with a guanine vacancy: G3VL1-9, or a bulge: G3bulge). **f**, **g** rG4-seq profiles of G2 G-rich region on *AT4G30460* (**f**) and G3 G-rich region on *AT3G23450* (**g**), otherwise in **c**. **h** The prevalence of both detected and computational-predicted G-rich regions in different genic regions. Computational-predicted G-rich regions were obtained by searching the sequence feature of GxLnGxLnGxLnGx in *Arabidopsis* transcriptome. **i** Comparison of base-pairing probability (BPP) of alternative secondary structure in G-rich regions that are detected with K^+^(blue) and undetected (grey) using rG4-seq. The Gs in the G-rich region were classed into 8 bins; flanking sequences (100 nt on both sides) were classed into 20 bins, with 15 bins close to G-rich regions shown. Differences of BPPs between G-rich regions and flanking regions, detected by rG4 with K^+^, *P* = 0.444; undetected regions, *P* < 10^−16^; *P* values, paired Student’s *t* test. **j** Secondary structure of G-rich region detected by rG4-seq (in **g**) and flanking sequences on *AT3G23450*, predicted using *Vienna RNAfold*. The filling colours of orange, green and blue indicate the base-pairing probability of below 0.3, 0.3–0.7 and above 0.7, respectively. Red stars indicate the guanines comprising the G3 region. Numbers indicate nucleotide positions on the transcript

To systematically search for G-rich regions with the potential to fold into RG4s in *Arabidopsis*, we used rG4-seq, an in vitro approach for detecting RG4 formation at a transcriptome-wide scale [25]. RG4 formation in vitro is stabilized by potassium ions (K^+^) (Fig. 1a), but not lithium ions (Li^+^), and is preferentially stabilized by G-quadruplex stabilizing ligands such as pyridostatin (PDS) [26]. G-rich regions which folded into RG4s in vitro can cause reverse transcriptional stalling (RTS) [21, 25]. Therefore, RTS sites dependent on the presence of K^+^ or K^+^+PDS suggest the presence of G-rich regions with RG4 folding potential within sequences upstream of the RTS (Fig. 1b). We performed reverse transcription on *Arabidopsis* RNAs with Li^+^, K^+^ or K^+^+PDS and generated corresponding libraries with high reproducibility (Additional file 1: Figure S1). To validate our rG4-seq, we mapped RT stops on the mRNA of *SUPPRESSOR OF MAX2 1-LIKE5* (*SMXL5)* which contains a G-rich region with RG4 folding potential identified recently [9]. We found a strong RT stalling at the 3′end of this G-rich region where the coverage dropped in the presence of both K^+^ and K^+^+PDS conditions, but not Li^+^ (Fig. 1c), agreeing with the previous gel-based assay [9].

We then searched for RTS sites dependent on the presence of K^+^ or K^+^+PDS in the *Arabidopsis* transcriptome. Our meta-analysis showed that guanine (G) was strongly enriched in sequences upstream but not downstream of RTS sites, for both K^+^ and K^+^+PDS conditions (Fig. 1d). A strong enrichment of guanine suggested the prevalence of G-rich regions with the potential to form RG4s in vitro [17, 25]. We searched for G-rich regions able to form RG4s on the basis of special sequence feature, GxLnGxLnGxLnGx (whereby G stands for guanine; L stands for loop, see the “Methods” section) in sequences upstream of RTS. In total, we found 381 and 2457 G-rich regions with strong RTS dependent on K^+^ and K^+^+PDS, respectively (Additional file 2: Table S1, Table S2). We then classified these G-rich regions according to G-quartet number, loop length and bulge size. In the presence of K^+^, we found 253 G2 (with two G-quartets) and 128 G3 (with three G-quartets) G-rich regions (Fig. 1e and Additional file 2: Table S1). In the presence of K^+^+PDS, we detected 2234 G2 and 223 G3 regions (Fig. 1e and Additional file 2: Table S2). As illustrated by rG4-seq profiles of individual regions on *AT4G30460* and *AT3G23450*, coverage dropped down in the presence of K^+^ and K^+^+PDS, but not Li^+^, at the 3′end of these G-rich regions, indicating folding of G2 and G3 RG4s (Fig. 1f, g). We also examined the location of both detected G-rich regions and computational-predicted G-rich regions and found that they were mostly localized in the coding regions (Fig. 1h and Additional file 2: Table S1, Table S2). Taken together, we revealed the global in vitro landscape of G-rich regions with the potential to fold into RG4s in the *Arabidopsis* transcriptome.

### Features of *Arabidopsis* RG4 formable regions

Over 65,000 G-rich regions were predicted in silico to form RG4s in *Arabidopsis* based on the sequence feature of GxLnGxLnGxLnGx [6, 27]. However, we detected less than 3000 regions by rG4-seq (Fig. 1e and Additional file 2: Table S1, Table S2), indicating most of the predicted regions are unlikely to fold into RG4s in vitro. This prompted us to ask whether this low rate of in vitro folding is due to the competition from alternative secondary structure formation, which might prevent RG4 formation across these predicted regions. To test this hypothesis, we used the *Vienna RNAfold* software to predict the secondary structures of both in silico predicted but undetected G-rich regions and the detected RG4 regions by rG4-seq with K^+^ [28]. We then calculated the base-pairing probability (BPP) of each nucleotide based on the predicted secondary structures and performed the meta-property analysis [28]. We found that, for the undetected G-rich regions, the BPPs of the G-rich regions were significantly higher compared to the flanking regions (Fig. 1i, *P* < 10^−16^, paired Student’s *t* test), indicating these G-rich regions were folded into strong secondary structures. In contrast, for those detected G-rich regions in the presence of K^+^, no significant differences of BPPs were found between G-rich regions and flanking sequences (Fig. 1i, *P* = 0.444, paired Student’s *t* test), and the BPPs of G-rich regions are strongly lower than that of the undetected regions (Fig. 1i, *P* < 10^−16^, Student’s *t* test). Therefore, those regions detected in vitro by rG4-seq are likely to form weak Watson-Crick-based secondary structure while the undetected regions are likely to fold into strong Watson-Crick-based secondary structures (Fig. 1j and Additional file 1: Figure S2). Taken together, our results indicate that alternative secondary structures in those G-rich regions strongly affect the potential of folding into RG4s in vitro.

### SHALiPE-Seq robustly determines the folding state of G-rich regions in vitro

Next, we asked if these G-rich regions with folding potential in vitro are able to fold into RG4s in vivo. Our previous studies have shown that both NAI and DMS are capable of penetrating plant cells [29, 30]. We selected NAI rather than DMS for our in vivo determination of the folding state of the G-rich regions in plants, because the DMS method using high DMS concentration (8%), causes plant wilting as well as significant RNA decay [17, 31]. To avoid unpredictable inaccuracies of structure determination implied by the high concentration of DMS method [32], we employed the NAI chemical probing method by coupling our previous method selective 2′-hydroxyl acylation with lithium ion-based primer extension (SHALiPE) with high-throughput sequencing, which we termed SHALiPE-Seq [19, 29].

**Fig. 2:**
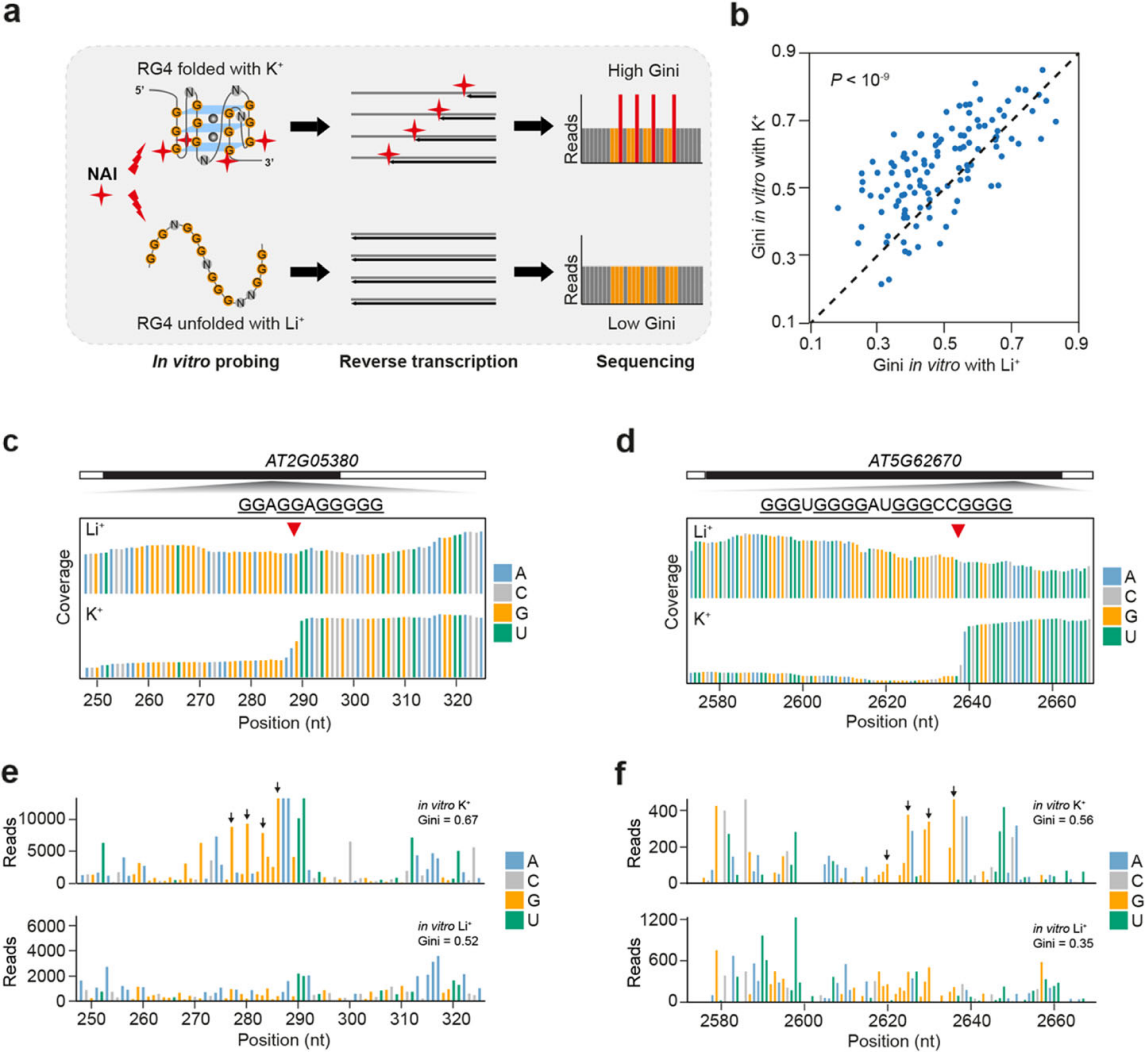
SHALiPE-Seq determines folding states of G-rich regions robustly*.* **a** Schematic of SHALiPE-Seq in vitro with K^+^ and Li^+^. In vitro probing in the presence of K^+^ and Li^+^ established the benchmarks of folded and unfolded states of G-rich regions, respectively. NAI (indicated by red cross) preferentially modifies the last G in G tracts of folded RG4s, resulting in a high Gini index of read counts (reads on preferentially modified Gs were in red) in SHALiPE profiles. In contrast, the distribution of the SHALiPE profile for the unfolded state in the presence of Li^+^ is uniform, resulting in a low Gini. **b** For G-rich regions detected using rG4-seq in the presence of K^+^, Gini of SHALiPE profiles in vitro with K^+^ (folded state) was greater than that of in vitro with Li^+^ (unfolded state) by a factor of 1.20 (*n* = 117, *P* value, paired Student’s *t* test, average read coverage on G ≥ 50 reads/nt). **c**, **d** rG4-seq profiles of G2 G-rich region on *AT5G05380* (**c**) and G3 G-rich region on *AT5G62670* (**d**). The 3′end of the G-rich region is indicated by a red triangle. A (blue), C (light grey), G (yellow) and U (green). **e**, **f** SHALiPE profiles in vitro with K^+^ or Li^+^ of G2 G-rich region on *AT5G05380* (**e**) and G3 G-rich region on *AT5G62670* (**f**). High read counts of last guanines of G-tracts (indicated by black arrows) represent strong modifications of NAI on these guanines in the presence of K^+^, indicating RG4s are folded. In the presence of Li^+^, last Gs are not strongly modified, representing the unfolded state of these G-rich regions. The Gini values with K^+^ are higher than those with Li^+^, as indicated. A (blue), C (light grey), G (yellow) and U (green)

SHALiPE-Seq is based on the preferential modification of the last G in G tracts of folded RG4s by 2-methylnicotinic acid imidazolide (NAI) (Fig. 2a) [17, 19, 33]. Strong modifications are able to cause reverse transcription stalling on these last guanines, which are detectable by deep sequencing. Significantly higher reads number can be found on these last guanines for folded G-rich regions, rather than unfolded ones (Fig. 2a). This method could discriminate the folded state from the unfolded state of individual G-rich regions by showing reads number with or without the special pattern. Before applying this method to plants in vivo, we firstly established benchmark SHALiPE profiles for both folded and unfolded states of G-rich regions in plants in vitro in the presence of K^+^ (folded state) or Li^+^ (unfolded state), respectively (Fig. 2a, see the “Methods” section). We assured our NAI modification efficiencies in the presence of K^+^ and Li^+^ were similar, as indicated by gel-based analysis on 18S rRNA (Additional file 1: Figure S3A). We then generated the corresponding SHALiPE-Seq libraries with high reproducibility (Additional file 1: Figure S3B-C).

In these SHALiPE-Seq libraries, the distribution of read counts on guanines for the folded state (with K^+^) is uneven, while the distribution for the unfolded state (with Li^+^) is uniform (Fig. 2a) [17]. These distributions were measured using the Gini index, where a high Gini indicates an uneven distribution (folded state) and a low Gini indicates a uniformed distribution (unfolded state) (Fig. 2a) [17]. As expected, for the G-rich regions detected by rG4-seq, the Gini of SHALiPE profiles in vitro with K^+^ was greater than that in vitro with Li^+^ by a factor of 1.20 (Fig. 2b, *P* < 10^−9^, paired Student’s *t* test and Additional file 2: Table S3, reads ≥ 50/nt). As illustrated by the individual G2 and G3 regions on *AT2G05380* and *AT5G62670* identified by rG4-seq (Fig. 2c, d), high read counts were found on the last Gs (indicated with black arrows) in the SHALiPE profiles with K^+^, indicating the preferential NAI modification on these Gs when RG4s are formed (Fig. 2e, f, top channels). Conversely, read counts in the SHALiPE profile with Li^+^ are more uniform, representing the unfolded state of these G-rich regions (Fig. 2e, f, bottom channels). As a result, the Ginis of the SHALiPE profiles in the presence of K^+^ (0.67 and 0.56) are higher compared to the corresponding 0.52 and 0.35 for Li^+^ (Fig. 2e, f, text annotation on the upper right). Further, we calculated Gini on the Gs not in clusters and Gini on the other bases (A, C and U) of the SHALiPE profiles in the presence of Li^+^ or K^+^, respectively. No significant differences between Gini with Li^+^ and Gini with K^+^ were found (Additional file 2: Figure S4), supporting the strong specificity of SHALiPE-seq method for RG4 detection. These results indicate that SHALiPE-Seq and rG4-seq are in strong mutual agreement and that SHALiPE-Seq profiling is able to determine the folding state of individual G-rich regions at the transcriptome-wide scale.

### Stable folding of RG4s in *Arabidopsis* in vivo

**Fig. 3:**
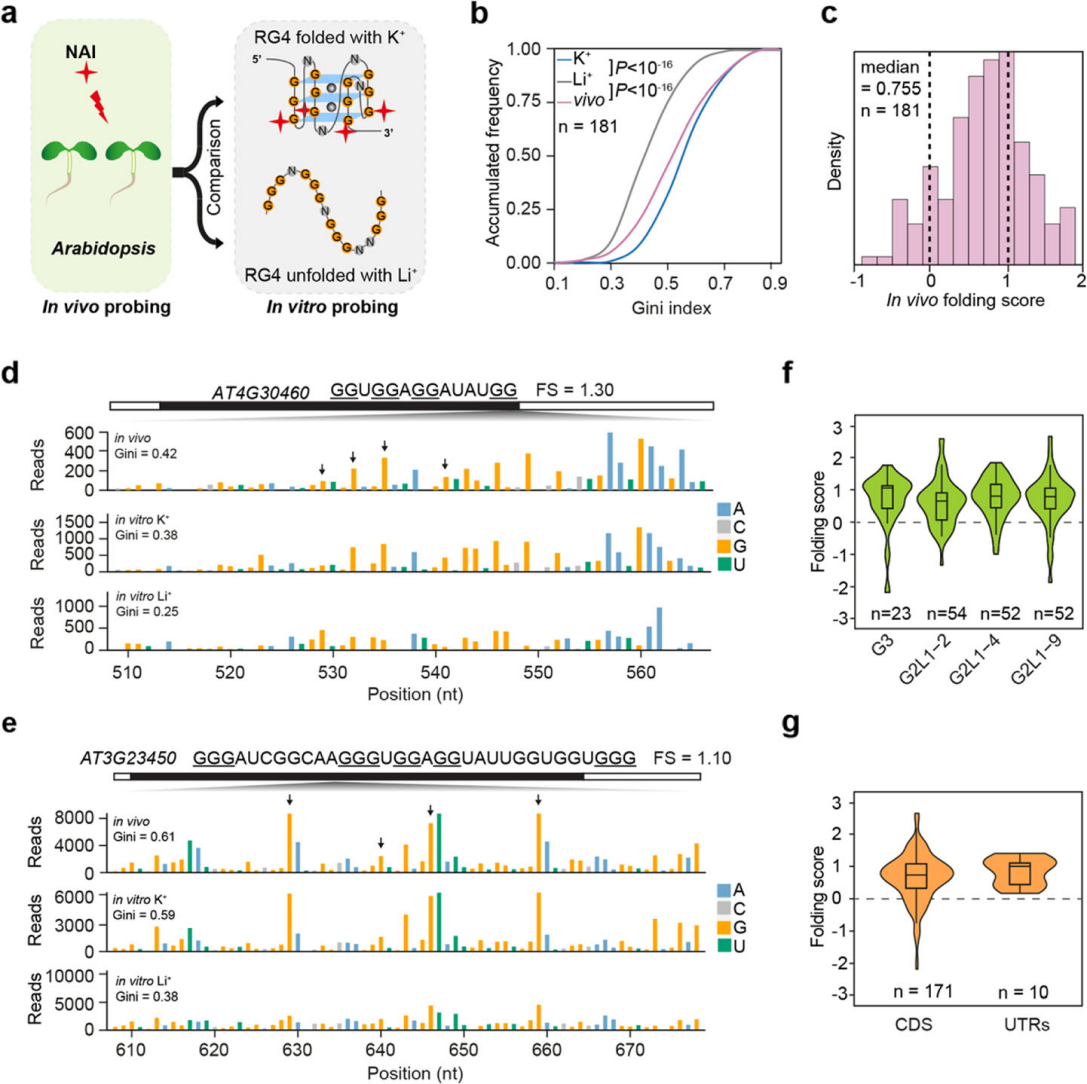
In vivo SHALiPE-Seq reveals hundreds of folded RG4s in *Arabidopsis.* **a** Schematic of in vivo SHALiPE-Seq in *Arabidopsis*. In vivo folding state of G-rich region is evaluated by comparing SHALiPE profiles in vivo with SHALiPE profiles in vitro in the presence of Li^+^ or K^+^, respectively. **b** Cumulative plot of Gini index of *Arabidopsis* G-rich regions in vivo, in vitro with Li^+^ and in vitro with K^+^. A significantly higher Gini in vivo than that in vitro with Li^+^ indicates the folding state of G-rich regions in vivo. *P* value, paired Student’s *t* test. **c** Histogram of in vivo folding score (FS) in *Arabidopsis.* The median value is 0.755. The FS of 0 represents the unfolded states (with Li^+^) and 1 represents the folded states of RG4s (with K^+^) in vitro*.* **d**, **e** SHALiPE profiles of the RG4 region on *AT4G30460* (**d**) and *AT3G23450* (**e**). The in vivo SHALiPE profile resembled the in vitro SHALiPE profile with K^+^ (the last Gs of G-tracts indicated by black arrows) but not the in vitro SHALiPE profile with Li^+^, indicating the folded state of this RG4 in vivo. **f** Violin plot of in vivo folding scores of G-rich regions of different types. Folding scores of RG4s of different catalogues are similar (*P* values > 0.05, one-way ANOVA/Tukey HSD post hoc test). **g** Violin plot of in vivo folding scores of G-rich regions in CDS and UTRs. Folding scores of RG4s of different genic regions are similar (*P* > 0.05, one-way ANOVA/Tukey HSD post hoc test)

To assess the in vivo folding state of these G-rich regions, we firstly performed in vivo NAI chemical probing in *Arabidopsis* [29] and generated in vivo SHALiPE-Seq libraries with high reproducibility (Additional file 1: Figure S3D). We then compared SHALiPE profiles in vivo with our benchmark SHALiPE profiles in vitro for both folded and unfolded states (Fig. 3a) [17, 19, 29]. Next, we calculated the Gini in vivo of G-rich regions and found that Gini in vivo was greater than that in vitro with Li^+^ by a factor of 1.10 (Fig. 3b, *P* < 10^−16^, paired Student’s *t* test and Additional file 2: Table S4, reads ≥ 50/nt). This result suggested that these G-rich regions are folded into RG4s in vivo. To further quantify the folding state in vivo, we calculated the in vivo folding score: a comparison of Gini in vivo with Gini in vitro in the presence of Li^+^, scaled relative to Gini in vitro with K^+^ vs Gini in vitro with Li^+^ [17]. Out of the 181 G-rich regions, in vivo folding scores for 153 regions were over 0 and centred near 1 with a median value of 0.755 (Fig. 3c, Additional file 1: Figure S5A-B and Additional file 2: Table S4), indicating that most G-rich regions identified by rG4-seq were strongly folded in *Arabidopsis*. The in vivo folding of RG4s in *Arabidopsis* differs from the unfolded states in yeast and mouse embryonic stem cells, where the folding scores centred near 0 (median values of − 0.02 and 0.06, respectively) [17]. The in vivo SHALiPE profile resembled the in vitro SHALiPE profile in the presence of K^+^ (folded state), but not in the presence of Li^+^ (unfolded state) for individual G-rich regions, as exemplified by the regions on *AT4G30460* and *AT3G23450* (Fig. 3d, e), which showed a K^+^-dependent coverage drop of rG4-seq profile (Fig. 1f, g). We further assessed whether any specific type of G-rich regions may be preferentially folded in vivo, but found very similar folding scores amongst different types of RG4s indicating no specific preference (Fig. 3f and Additional file 1: Figure S5C). In addition, the folding scores for RG4s in both coding regions (CDS) and untranslated regions (UTRs) were quite similar (Fig. 3g), indicating both CDS and UTRs contain stable RG4s in vivo. Taken together, our results indicate that in *Arabidopsis*, hundreds of G-rich regions are strongly folded into RNA G-quadruplexes in vivo, unlike the previously reported in vivo observations in yeasts and mice [17].

### In vivo folding of RG4s in rice

**Fig. 4:**
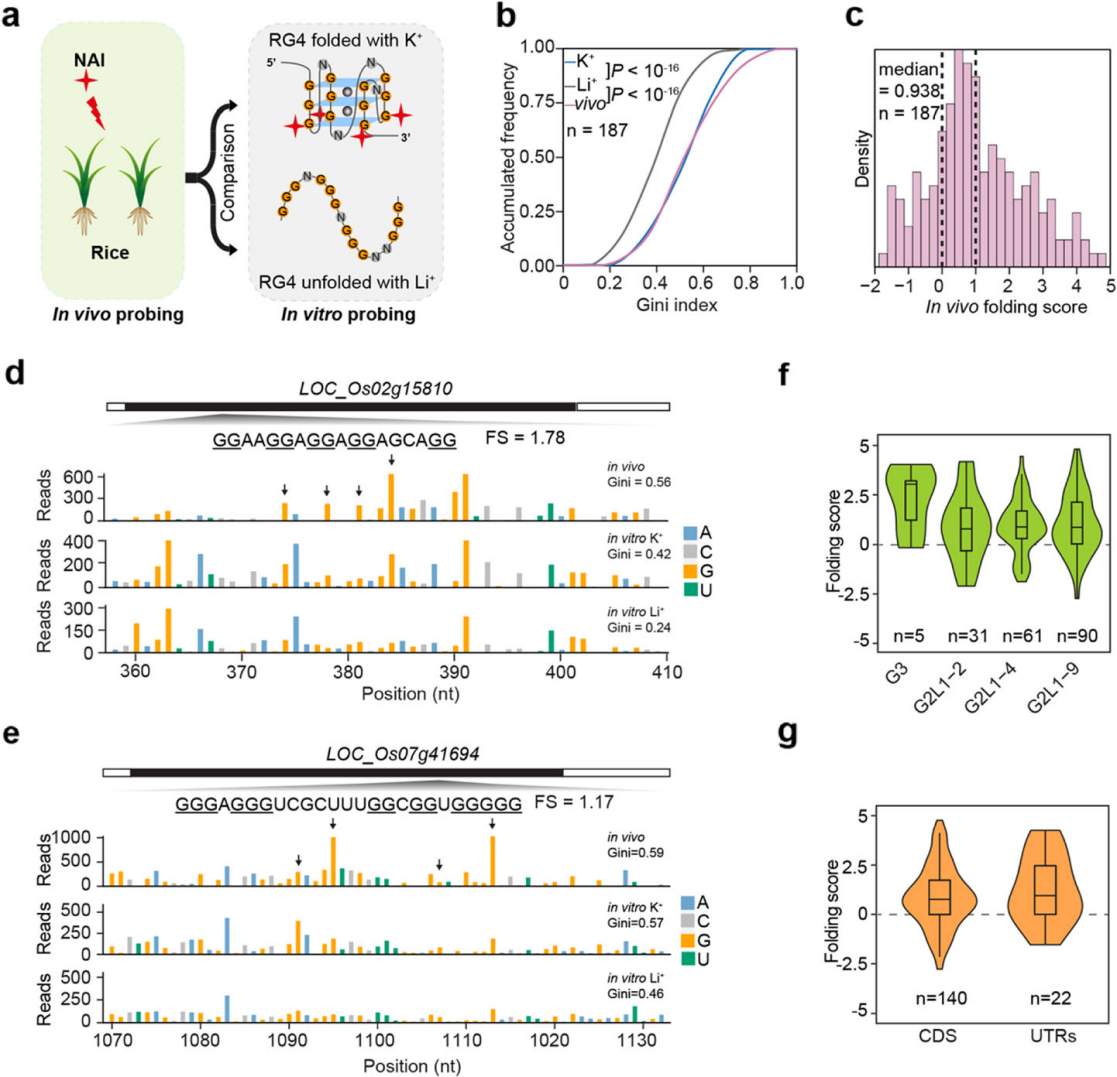
In vivo SHALiPE-Seq reveals hundreds of folded RG4s in rice. **a** Schematic of in vivo SHALiPE-Seq in rice. The in vivo folding state of the G-rich region is evaluated by comparing SHALiPE profiles in vivo with SHALiPE profiles in vitro in the presence of Li^+^ or K^+^, respectively. **b** Cumulative plot of Gini index of rice G-rich regions in vivo, in vitro with Li^+^ and in vitro with K^+^. A significantly higher Gini in vivo than in vitro with Li^+^ indicates the folding state of G-rich regions in vivo. *P* value, paired Student’s *t* test. **c** Histogram of in vivo folding score (FS) in rice*.* The median value is 0.938. The FS of 0 represents the unfolded states (with Li^+^) and 1 represents the folded states of RG4s (with K^+^) in vitro*.* **d**, **e** SHALiPE profiles of the RG4 region on *LOC_Os02g15810* (**d**) and *LOC_Os07g41694* (**e**). The in vivo SHALiPE profile resembled the in vitro SHALiPE profile with K^+^ (the last Gs of G-tracts indicated by black arrows) but not the in vitro SHALiPE profile with Li^+^, indicating the folded state of this RG4 in vivo. **f** Violin plot of in vivo folding scores of G-rich regions of different types. Folding scores of RG4s of different catalogues are similar (*P* values > 0.05, one-way ANOVA/Tukey HSD post hoc test). **g** Violin plot of in vivo folding scores of G-rich regions in CDS and UTRs. Folding scores of RG4s of different genic regions are similar (*P* > 0.05, one-way ANOVA/Tukey HSD post hoc test)

To determine whether RG4s exist in diverse plant species, we investigated the folding state of G-rich regions in rice (*Oryza sativa* ssp. *japonica*), one of the most globally important crops [34]. We performed SHALiPE-Seq profiling in rice and compared in vivo SHALiPE profiles with in vitro with K^+^ and Li^+^, respectively (Fig. 4a). Similar to the observations in *Arabidopsis*, Gini in vivo for the 187 regions in rice was greater than that in vitro with Li^+^ by a factor of 1.10 (Fig. 4b, *P* < 10^−16^, paired Student’s *t* test, and Additional file 2: Table S5). In vivo folding scores centred near 1 with a median value of 0.938, indicating the folding status of RG4s in rice (Fig. 4c, and Additional file 2: Table S5). The in vivo SHALiPE profile resembled the in vitro SHALiPE profile in the presence of K^+^ (folded state) but not Li^+^ (unfolded state) for individual RG4s, as exemplified by the regions on *LOC_Os02g15810* and *LOC_Os07g41694* (Fig. 4d, e). Moreover, no significant differences were found of the folding scores for RG4s of specific types nor different genic locations (Fig. 4f, g). Taken together, as represented by model dicot and monocot plant species, our results suggest the general existence of RG4s in the plant kingdom. Interestingly, there are 121 orthologue gene pairs containing detected RG4s in *Arabidopsis* and rice (Additional file 2: Table S6), suggesting RG4s are likely to be conserved in plants. We then performed a Gene Ontology (GO) analysis using the transcripts containing RG4s in *Arabidopsis* and rice. We found that the enriched GO items were highly overlapped between *Arabidopsis* and rice, particularly in the molecular functions related to RNA binding and protein binding (Additional file 1: Figure S6), implying that these RG4s may be involved in conserved functions in plants.

### RG4 regulates translation and plant development

**Fig. 5:**
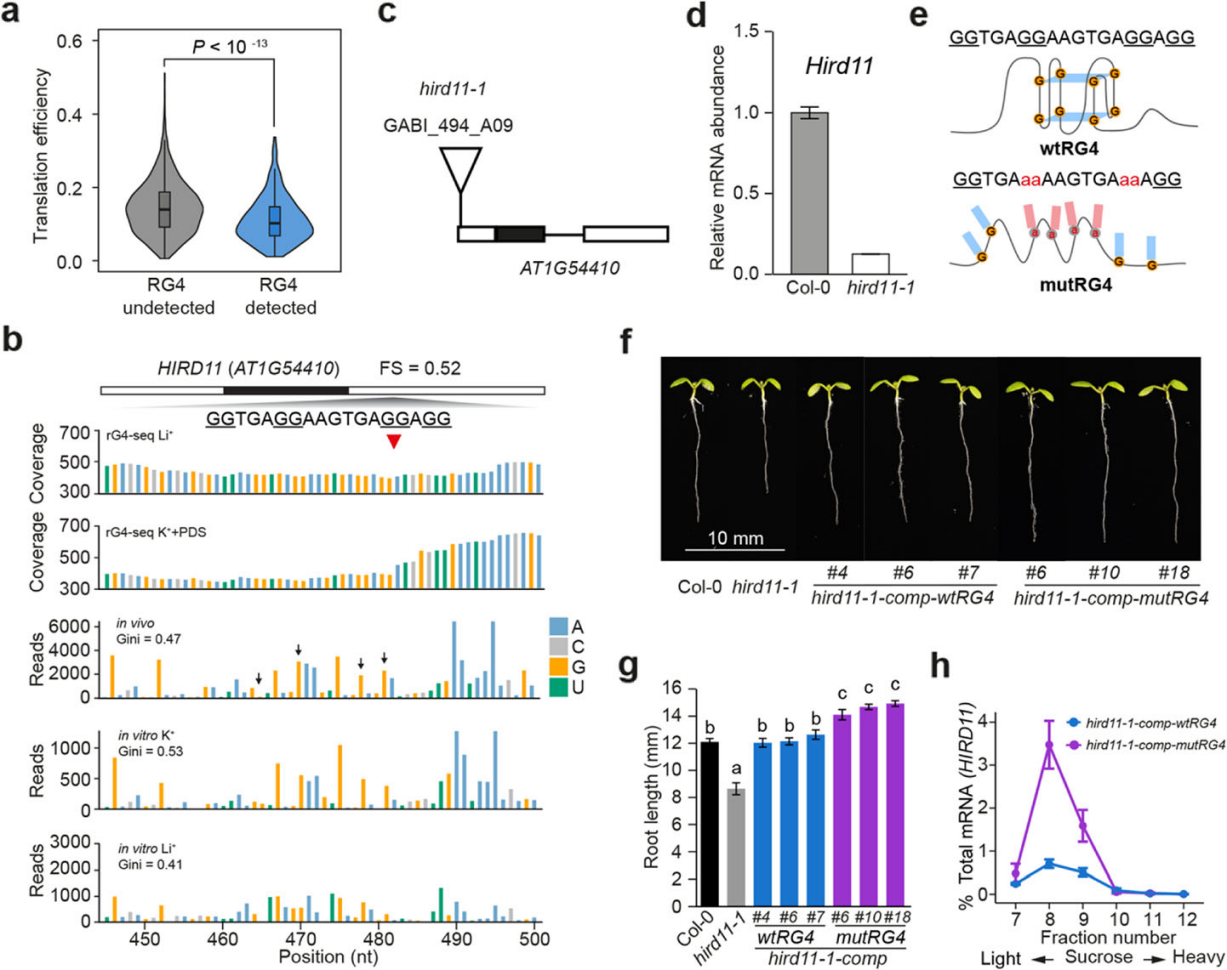
RG4 regulates plant growth and translation. **a** Violin plot of translation efficiency (TE) of transcripts for predicted but undetected RG4s (RG4 undetected) and detected RG4s (RG4 detected) using rG4-seq. TE of transcripts with detected RG4s is significantly lower than that of RG4 undetected transcripts (*P* < 10^−13^, Student’s *t* test). **b** rG4-seq and SHALiPE profiles of the RG4 region on 3′UTR of *HIRD11* (*AT1G54410*). The in vivo SHALiPE profile resembled the in vitro SHALiPE profile with K^+^ (the last G in G tracts of the RG4 region indicated by black arrows) but not the in vitro SHALiPE profile with Li^+^, indicating the folded state of this RG4 in vivo. **c** Schematic diagram of *HIRD11* showing the T-DNA insertion site of *hird11-1* (GABI_494_A09). **d** Relative mRNA abundance of *HIRD11* in Col-0 and *hird11-1* plants indicated the *hird11-1* is a knock-down mutant; error bars indicate SE. **e** Sequences of wild-type RG4 (wtRG4, left) and disrupted G-rich region with G to A mutation (mutRG4, right) on *HIRD11*. **f**, **g** RG4 modulates plant growth. Representative 6-day-old plants of Col-0, *hird11-1*, complemented *hird11-1* with wild-type RG4 (*hird11-1-comp-wtRG4*) and complemented *hird11-1* with mutated RG4 (*hird11-1-comp-mutRG4*) (**f**), and average primary root lengths (**g**) of more than 20 plants for each genotype, 3 representative lines out of 6 independent lines for wtRG4 plants and 9 independent lines for mutRG4 plants were shown. Significant differences were evaluated by one-way ANOVA/Tukey HSD post hoc test (*P* < 0.05). Error bars indicate SE. **h** Analysis of polysome-associated *HIRD11* mRNA in the transgenic plants. RNA abundance of *HIRD11* in each polysome associated fraction was detected by qRT-PCR and quantified as a percentage relative to their total amount, error bars indicate SE

Several previous studies suggested that RG4s may be associated with translational regulation of gene expression in *Arabidopsis* [9, 24]. To test whether RG4s generally affect translation, we reanalyzed the ribosome profiling data in *Arabidopsis* and calculated the translation efficiency (TE) of transcripts containing either detected RG4s or undetected RG4s [35]. We found that TEs of those mRNAs containing folded RG4s are significantly lower than those without folded RG4s (Fig. 5a, *P* < 10^−13^, Student’s *t* test), suggesting that folded RG4s tend to have a general effect of repressing translation efficiency of mRNAs.

To validate the global association between RG4s and their regulatory functions, we further investigated the functional role of individual RG4. We focused on those in vivo folded RG4s (folding score > 0.5) located in UTRs of genes in *Arabidopsis* (Additional file 2: Table S4). We screened T-DNA mutants of these genes and successfully identified homozygotes for gene *HIRD11*, which encodes a KS-type dehydrin and contains a G2 RG4 in its 3′UTR (Fig. 5b, c and Additional file 1: Figure S7A). The mRNA abundance of *HIRD11* in the mutant (termed *hird11-1*) was strongly reduced compared to wild-type Col-0 (Fig. 5d). The growth of *hird11-1* was largely retarded relative to Col-0, represented by shorter primary roots compared to Col-0 (Fig. 5f, g). The phenotype of shorter primary roots in *hird11-1* was restored by complementing mutants with *HIRD11* containing wild-type RG4 sequence (wtRG4), indicating *HIRD11* promotes plant growth (Fig. 5e–g). Strikingly, when *hird11-1* mutant was complemented with *HIRD11* containing mutated RG4 sequence with four G residues substituted by A residues (mutRG4), which excludes the possibility of RG4 folding (Fig. 5e), the primary root length of three independent lines of mutRG4 plants was distinctively longer than that of wtRG4 plants (Fig. 5f, g).

We assessed whether RG4 folding affected the gene expression of *HIRD11*. Firstly, we compared the mRNA abundance of *HIRD11* in wtRG4 and mutRG4 plants and found no significant difference in mRNA between these plants (Additional file 1: Figure S7B). We then determined whether RG4 folding may affect *HIRD11* translation. Since no commercial antibody against HIRD11 is available, we used polysome analysis by combining sucrose gradient fragmentation with qRT-PCR to detect the translational level of *HIRD11* in these plants [36, 37]. We found that polysome-associated *HIRD11* mRNA in mutRG4 plants was much higher than that in wtRG4 plants (Fig. 5h), indicating a higher translational level of HIRD11 in mutRG4 plants. Furthermore, we assessed the effect of this RG4 on repressing translation using the dual-luciferase reporting system, by fusing the *HIRD11* 3′UTR containing wtRG4 or mutRG4 with the coding region of Firefly luciferase reporter gene (Additional file 1: Figure S8A). Firefly luciferase activity (F-LUC) of mutRG4 was ~ 3 times higher to that of wtRG4, when normalized to the Renilla luciferase activity (R-LUC) (Additional file 1: Figure S8B, *P* < 10^−11^, Student’s *t* test). We also measured RNA abundance and found no significant differences between wtRG4 and mutRG4 (Additional file 1: Figure S8C). Therefore, our results indicate the RG4 on *HIRD11* suppresses its translation to modulate plant growth and development.

## Discussion

‘If G-quadruplexes form so readily in vitro, nature will have found a way of using them in vivo’ (statement by Sir Aaron Klug over 30 years ago) [38]. Following decades of research by the community exploring RG4 across living eukaryotic cells, here for the first time, we determined hundreds of RG4s folded in model dicot and monocot plant species, providing direct evidence of RG4 existence in eukaryotes (Figs. 3 and 4). By both genetic and biochemical validations, we also demonstrated the important roles of the RG4 in modulating plant growth and development (Fig. 5).

### Unique landscape of RNA G-quadruplex formable regions in *Arabidopsis*

RG4s contain unique sequence features of GxLnGxLnGxLnGx (whereby G stands for guanine, L stands for loop, and *x* ≥ 2, *n* up to 15). For decades, in silico prediction based on sequence features was used to search for putative RG4s at the genome-wide scale in different organisms [6, 27, 39, 40]. With the rise of deep sequencing methods, high-throughput methods such as rG4-seq were developed to map G-rich regions with in vitro RG4 folding potential throughout the transcriptome [25]. Here, we performed rG4-seq to map these G-rich regions in the *Arabidopsis* transcriptome. We identified less than 3000 G-rich regions with RG4 folding potential in vitro (Additional file 2: Table S1, Table S2), which is much less than over 65,000 G-rich regions predicted using the in silico sequence-based method [27]. Following our assessment of RNA secondary structures, we found that alternative secondary structures within those G-rich regions folded into compete with RG4 formation in the transcriptome (Fig. 1i, j), agreeing with the previous discoveries on a single example of G_4_C_2_ repeats in amyotrophic lateral sclerosis (ALS)-associated gene *C9ORF72* [41]. If the G-rich region is able to form a strong secondary structure, it is unlikely to fold into RG4. This might be due to the slower folding kinetics for RG4 formation compared to the formation of alternative secondary structures [42]. Thus, from a transcriptome-wide perspective, RG4 folding capability is generally influenced by alternative secondary structure formation, which may be an important way of regulating RG4 folding potential. Our results suggested that apart from the sequence feature of G-rich regions, the secondary structure of the flanking regions should be considered in predicting putative RG4 sites.

Following our rG4-seq in vitro profiling, we also revealed unique features for G-rich regions with RG4 folding potential in plants. We found that G-rich regions with potential to form G2 RG4s rather than G3 RG4s are predominate (> 90%) in plants (Fig. 1e and Additional file 2:Table S1, Table S2), whereas in human cells, G-rich regions with potential to fold G3 RG4s are dominant [25]. Rather than the very stable structures that G3 RG4s fold (Tm > 55 °C), G2 RG4s are less stable (Tm ~ 14–30 °C) [43, 44], thereby harbouring higher flexibility to switch between folded and unfolded states within the temperature range that most plants favour. This flexibility may facilitate a regulatory role in plant adaption to the immediate local environment. Rather than the depletion of G-regions for folding into RG4 in bacteria or avoiding the formation of stable G3 RG4s in animals [17], plants seem to have evolved a preference for G2 RG4 formation, which might be one of the reasons why plants have adopted RG4 as important regulators.

### In vivo folding of RNA G-quadruplex structures in plants

In our study, we specifically aimed to explore the existence of RG4 in vivo. Therefore, it is critical to first set up the standards to confidently distinguish the unfolded and folded status of G-rich regions, for subsequent evaluation of in vivo folding status. To do this, we used two in vitro methods (rG4-seq and SHALiPE-seq in vitro) to provide confident assurance of the correct SHALiPE profiles for in vitro folded RG4s. We then compared the in vivo SHALiPE profile with the SHALiPE profiles for in vitro folded RG4s. We set up very strict criteria for our comparative in vitro and in vivo data analyses, such as using very high sequencing depth cut-off (RT stop read count ≥ 50 for each G). Therefore, it is possible that there may be more in vivo folded RG4s than the number we reported. The large number of RG4s present in plants (Figs. 3 and 4) is probably explained by the physiological conditions in plants being favourable towards RG4 formation. For example, potassium (K^+^) is the predominant cytoplasmic inorganic cation in plants with a concentration of around 100 mM [45, 46], which is preferable for RG4 folding. Notably, unlike animals, plants lack a potassium/sodium exchanger and thus use a unique accumulation and release system for potassium [46]. Since RG4 folding is highly sensitive to potassium levels, RG4s have great potential to be adopted as a regulator in response to dynamic potassium level changes. Our gene ontology analysis (Additional file 1: Figure S6A) confirmed that genes involved in metal ion binding are significantly enriched (Additional file 1: Figure S6A). As such, RG4s might contribute to maintaining an optimum potassium level in plants. Apart from potassium levels, temperature is another key factor that was suggested to affect the folding of RG4s in vitro [43, 44]. The optimal environmental temperature for plants (21–22 °C for *Arabidopsis* and 26–28 °C for rice) is much lower than the body temperature of animals (37 °C for human and 36.6 °C for mice). Thus, this relatively low temperature may allow the stable formation of RG4s in plant cells. In addition, a recent study found that the *Arabidopsis* RNA-binding protein, zinc-finger protein JULGI, preferentially binds to a specific G-rich sequence with in vitro folding potential, stabilizing the RG4 in vitro even in the absence of potassium [9]. This result indicated that co-factors such as RNA-binding proteins might be important regulators for the formation of RG4s in plants. Notably, in vivo folding scores of many individual regions (60 out of 181 in *Arabidopsis*, ~ 1/3) are over 1 (Fig. 3c and Additional file 2: Table S4), indicating the in vivo folding state could be stronger than the in vitro folding state in the presence of K^+^. This result suggested that other factors might also promote RG4 formation in vivo. Hence, the ideal physiological conditions in plants together with those co-factors may confer plants with the ability to adopt RG4s, as regulators for gene expression.

### Different folding status of RNA G-quadruplex structures in living eukaryotes

Another interesting observation from our study is the variable folding states between *Arabidopsis* and rice (Figs. 3c and 4c). The distribution of in vivo RG4 folding scores in rice was shifted more to 1 compared to *Arabidopsis* (Figs. 3c and 4c). More RG4s in rice showed folding scores over 1 compared to the RG4s in *Arabidopsis* (Additional file 2: Table S4, Table S5). This result suggests that the folding landscape of RG4s is likely to be unique in different organisms under different growth conditions. Although previous chemical profiling in animal cell lines showed that RG4s are globally unfolded [17], one possible explanation could be that the conditions of the certain cell types tested by Guo and Bartel are not favourable for RG4 formation. Given the suggested functions of RG4s in special biological relevance, such as cancer cell growth and neurodegenerative diseases [7, 8, 18, 47, 48], RG4s might be able to form in specific cell types and growth conditions.

### Regulatory roles of RNA G-quadruplex structures in plant development

Previously, without direct evidence for RG4 formation in eukaryotes, it was not possible to infer whether suggested functions such as translation inhibition and splicing regulation are due to RG4 formation or specific sequence content [8, 9, 49, 50]. In our study, we selected an in vivo folded RG4 located on *HIRD11* to assess the functional impact of this RG4 (Fig. 5). The compelling phenotypic difference in root length between plants with and without RG4 indicated the significant impact of RG4 on plant development (Fig. 5). The direct evidence of in vivo RG4 formation substantiated by both genetic and biochemical validations provides the first demonstration of RG4 functionality in vivo. Given the large number of RG4s present in plants (Figs. 3 and 4), further research is warranted to demonstrate that RG4s could significantly influence many aspects of plant growth and development. Apart from the translational regulation we revealed here (Fig. 5), other biological functions such as splicing regulation might be also associated with RG4s [50]. Given the potential effect of sequence change in the mutated RG4s that may affect other post-transcriptional regulations of gene expression, more mutations could be considered in future studies for assessing the biological function of individual RG4s. Moreover, further exploration of the diverse functional roles of RG4s will expand our knowledge of RG4-dependent regulation of gene expression.

## Conclusions

Using in vitro and in vivo chemical RNA structure profiling, we determined the folding state of RNA G-quadruplex in living plants. In both *Arabidopsis* and rice, hundreds of RNA G-quadruplex structures are folded in vivo. The genetic validations revealed that the RNA G-quadruplex is able to regulate gene expression and to modulate plant development and growth. Our work, for the first time, gave a decided answer to the long-standing question that whether RNA G-quadruplex structures exist in living eukaryotic cells. Given the presence of a large number of RNA G-quadruplex structure in plants, the next step is to unravel their individual regulatory roles in plant growth, development and stress response.

## Methods

### Plants and growth conditions

The *Arabidopsis thaliana* ecotype of Columbia (Col-0), T-DNA insertion mutant GABI_494A09 (*hidr11-1*), was obtained from Nottingham *Arabidopsis* Stock Centre (NASC); homozygous mutants were identified by PCR-based genotyping [51]. *Arabidopsis* seeds were sterilized with 70% ethanol for 20 min, washed with distilled water for 3 times and plated on half-strength Murashige and Skoog medium with 1% (w/v) sucrose. After staying at 4 °C in dark for 3 days, the plates were placed in the growth chamber of 22 °C.

*Arabidopsis* mutants were transformed with the floral dipping method with the corresponding *Agrobacterium* [52]. Seeds of the transformed plants were plated to GM media with 10 mg/l phosphinotricin for the selection of transgenic plants.

### Plasmid construction

To complement the *Arabidopsis* mutants with wild-type (WT) sequence, DNA fragment of *AT1G54410* was amplified from Col-0 genomic DNA using CloneAmp HiFi PCR Premix (Clontech) with primers listed in Additional file 2: Table S7. To complement the *Arabidopsis* mutants with RG4-disrupted (mutRG4) sequence, fragments were amplified by overlap PCR with designed mutations (G to A) on primers [53] (Additional file 2: Table S7). PCR products were further introduced into the XmnI and KpnI digested pB7FWG2.0 with In-Fusion (Clontech). For dual-luciferase analysis, the sequence of 3′UTR of *AT1G54410* with or without mutation was cloned into the expression vector inter2 digested with AscI and PstI as introduced with designed primers (Additional file 2: Table S7). Sequencing-confirmed vectors were then transformed to *Agrobacterium tumefaciens* GV3101.

### rG4-seq library preparation

Libraries were generated according to our previous study [25]. Briefly, total RNA was extracted from etiolated seedlings of 5 days after germination of Col-0 with RNeasy Plant Mini Kit (Qiagen), followed by two rounds of poly(A) selection with polyA purist kit (Ambion). The poly(A)-selected RNA was fragmentated and ligated to 3′ adapter (5′/5rApp/AGATCGGAAGAGCACACGTCTG/3SpC3/3′). Ligated RNA was applied to reverse transcription with l μl 10 μM unlabeled reverse primer (5′CAGACGTGTGCTCTTCCGATCT3′) in the buffer (20 mM Tris (pH 8.3), 3 mM MgCl_2_, 1 mM DTT) containing 100 mM Li^+^ or 100 mM K^+^ or 100 mM K^+^+ 2 μM PDS. The cDNAs of 35–500 nt were purified and ligated to 5′ adapter (5′/5Phos/AGATCGGAAGAGCGTCGTGTAGCTCTTC-CGATCTNNNNNN/3SpC3/3′). Ligated cDNAs with the size of 70–500 nt were purified and applied to PCR reaction with a forward primer (5′AATGATACGGCGACCACCGAGATCTACACTCTTTCCCTACACGACGCTCTTCCGATCT3′) and reverse primer (5′CAAGCAGAAGACGGCATACGAGATNNNNNNGTGACTGGAGTTCAGACGTGTGCTCTTCCGATC3′, where NNNNNN denotes the barcodes, e.g. Index1 is CGTGAT for Illumina sequencing) and then submitted for next-generation sequencing by BGI (Hong Kong).

### In vitro NAI probing of RNA

For library construction, total RNA was extracted from 5-day etiolated seedlings of Col-0 using RNeasy Plant Mini Kit (Qiagen), followed by two rounds of poly(A) selection using poly(A) purist kit (Ambion); 1 μg poly(A)-selected total RNA in 50 mM Tris-HCl (pH 7.5), 0.5 mM MgCl_2_ and either 100 mM LiCl or 100 mM KCl was denatured at 95 °C for 90 s and cooled down on ice for 2 min. After sitting on 22 °C for 15 min, 2-methylnicotinic acid imidazolide (NAI) was added to a final concentration of 100 mM in 20 μl reaction, following with incubation for 5 min at 22 °C. NAI was quenched with 10 μl of 2 M DTT after incubation. The resulting reaction was loaded to Micro Bio-Spin® Columns with Bio-Gel® P-6 (BioRad) for cleaning up RNA, following with ethanol precipitation. For rice, total RNA was extracted from seedlings grown at 28 °C and applied to NAI probing at 28 °C.

### In vivo NAI probing of RNA

In vivo NAI probing was performed as described [29]. The NAI probing buffer was kept at 22 °C incubator overnight before use. For NAI probing at 22 °C, 5-day etiolated seedlings were harvested and incubated with 150 mM NAI for 15 min at 22 °C. Five times of DTT over NAI were immediately poured into the reaction to quench NAI with a vigorous vortex. The seedlings were ground in liquid nitrogen and applied to RNA extraction using the RNeasy Plant Mini Kit (Qiagen). For rice, seedling growth and in vivo NAI probing were performed at 28 °C.

### Gel-based analysis of NAI probing for 18S rRNA

One microgram of in vitro NAI-probed total RNA was dissolved in 6 μl water, and 1 μl of 5 μM Cy5-modified RT primer for 18S rRNA (listed in Additional file 2: Table S7) and 0.5 μl of 10 mM dNTPs were denatured at 95 °C for 3 min. The reaction was cooled down to 50 °C, 4 μl of 5X RT buffer (100 mM Tris (pH 8.3), 500 mM LiCl, 15 mM MgCl_2_, 5 mM DTT) and 0.5 μl Superscript III (Invitrogen) were added and mixed quickly with a pipette. The RT reaction was incubated at 50 °C for 20 min following a staying at 85 °C for 10 min to inactivate SSIII. cDNA-hybridized RNA was degraded by adding 0.5 μl of 2 M NaOH and incubation at 95 °C for 10 min. Equal volume of 2× stopping dye (95% formaldehyde, 20 mM EDTA (pH 8.0), 20 mM Tris (pH 7.5), orange G) was added and incubated at 95 °C for 5 min. The resultant reaction was kept on 65 °C and loaded to 8% acrylamide:bis-acrylamide-urea gel for electrophoresis. For the sequencing lanes, RNA was dissolved in 5 μl water; 1 μl 10 mM of corresponding ddNTP (Roche) was added at the beginning.

### Generation of the SHALiPE-Seq libraries

Libraries of SHALiPE-Seq were prepared as described [54]. In vitro or in vivo NAI probed, poly(A)-selected RNA was recovered and reverse transcribed using superscript III (Invitrogen) and RT primer (5′CAGACGTGTGCTCTTCCGATCTNNNNNN3′) with home-made RT buffer (20 mM Tris (pH 8.3), 100 mM LiCl, 3 mM MgCl_2_, 1 mM DTT). The 3′end of resultant cDNAs were ligated to a ssDNA linker (5′-PhosNNNAGATCGGAAGAGCGTCGTGTAG−/3SpC3/3′) using Circligase ssDNA Ligase (Epicentre) at 65 °C for 12 h. Ligated cDNA was applied to PCR amplification using KAPA Library Amplification Kits (Roche) with forward primer (5′AATGATACGGCGACCACCGAGATCTACACTCTTTCCCTACACGACGCTCTTCCGATCT3′) and reverse primer (5′CAAGCAGAAGACGGCATACGAGATNNNNNNGTGACTGGAGTTCAGACGTGTGCTCTTCCGATC3′, where NNNNNN denotes the barcodes, e.g. Index1 is CGTGAT for Illumina sequencing). The libraries were sequenced with Illumina HiSeq 4000 platform by BGI (Hongkong).

### Polysome-associated mRNA analysis

Polysome-associated mRNA analysis was performed as described [36, 37]. Briefly, around 500 mg 5-day etiolated seedlings was harvested and grounded into fine powder in liquid nitrogen; the powder was dissolved in 500 μl precooled polysome extraction buffer (200 mM Tris-HCl, pH 8.4, 50 mM KCl, 1% deoxycholic acid, 25 mM MgCl_2_, 2% Polyoxyethylene 10 tridecyl ether, 2 mM DTT, 400 U/ml recombinant Rnasin, 50 μg/ml cycloheximide) and incubated on ice for 30 min for sufficient lysis. After centrifugation at 13,200 rpm for 15 min at 4 °C, 500 μl supernatant was transferred to 15–60% sucrose gradient, following 4 h of centrifugation at 40,000 rpm in Beckman ST40Ti rotor. Fractions of 1 to 12 from low to high sucrose gradient were collected and conducted to RNA isolation with TRIzol reagent (Ambion). Fractions of 7 to 12 are associated with polysome [36, 37], representing the translation level. For RNA input of each sample, around 25-mg plant powder was applied to RNA isolation directly by RNeasy Plant Mini Kit (Qiagen).

### Quantitative real-time PCR

RNA was digested using RNase-free TURBO™ DNase (Ambion). First-strand cDNA was synthesized using Superscript III (Invitrogen) and oligo dT primer with home-made RT buffer (20 mM Tris (pH 8.3), 100 mM LiCl, 3 mM MgCl_2_, 1 mM DTT). Quantitative PCR was performed with LightCycler® 480 SYBR Green I Master (Roche) using CFX96 Touch Real-Time PCR Detection System (BIORAD) according to the manufacturer’s protocol. *PP2A* (*AT1G13320*) was used as the internal control for detecting the *HIRD11* expression in *Arabidopsis*. The expression level of the Renilla luciferase gene located on the same plasmid with the Firefly gene but in different transcription units was used as the internal control for measuring the transformation efficiency in tobacco leaves. Primers are listed in Additional file 2: Table S7.

### Phenotype assessment

To measure the primary root length, images of 6-day seedlings grown at 22 °C under long day (LD, 16/8 h, light/dark) were captured using a digital camera, and root lengths of more than 20 seedlings were measured using the ImageJ software (NIH).

### Dual-luciferase assay

Around 10 mg of leaf discs with 48 h of agroinfiltration were harvested and ground into fine powder in liquid nitrogen and homogenized in Passive Lysis Buffer (PLB, Promega), following with centrifugation at 13,000 rpm for 1 min. The clear supernatant was further diluted by 20 times with PLB and applied to dual luciferase assay using the Dual-Luciferase Reporter Assay System (Promega) according to the manufacturer’s manual.

### Alignment of the sequencing reads

For rG4-seq libraries, raw reads were directly used for alignment. For SHALiPE-Seq libraries, the first 3 bases at the 5′end (random nucleotides on adaptor for cDNA ligation) of the raw reads were trimmed before alignment. The reads were aligned against *Arabidopsis* transcriptome TAIR10 release using bowtie version 1.0.1with iterative mapping procedure [55]. The minimum read length allowed to map was fixed to 21 nt [56]. Up to three mismatches without any insertions or deletions were allowed to account for PCR and sequencing errors; only uniquely mapped reads were used for further processing [30, 54]. The resulting mapped sam files were converted to bam files and indexed using samtools-1.4.1 [57]. The stop counts were extracted using modules from HTseq v0.7.2 and pysam v0.11.2.2, and the code for analysis was written in Python v2.7.15. We merged the counts from replicates after observing high correlations between the replicates.

### Identification of RTS sites

To identify the regions where reverse transcription stalling (RTS) was strongly affected by the formation of RG4, we compared the read coverage and counts of rG4-seq in K^+^ vs Li^+^ and K^+^+PDS vs Li^+^ as described in our previous study [25]. Briefly, the bases of confident RTS were identified by comparison of read coverage between K^+^ vs Li^+^ and K^+^+PDS vs Li^+^, by fitting a linear model and estimate the *P* value of the fitting through ANOVA test, bases with *P* value smaller than 0.05 were selected.

### Hierarchical assignment of G-rich regions

Assignment of G-rich regions was performed according to our previous description [25]. For each RTS, sequences of 50 nt upstream the RTS site were extracted and assigned to different RG4s structural subclasses, defined as regular expressions used for pattern matching shown in square brackets:

G3L1-15: G3 RG4s with loop length between 1–15 nt [ G3N1-15+G3N1-15+G3N1-15+G3]

G3VL1-9: G3 RG4 with guanine vacancy (G3V) in one of the G-quartet layers, with loop length between 1 and 9 nt [G3N1-9 +G3N1-9+G3N1-9+G2 or G2N1-9+G3N1-9+G3N1-9+G3]

G3bulge: G3 RG4 with a bulge of loop, with loop length between 1 and 9 nt [G3N1-9+ (G2HGN1-9 or G2HGN1-9) + (G2HGN1-9 or G2HGN1-9)+G3]

G2L1-2: G2 RG4s with loop length between 1 and 2 nt [G2N1-2+G2N1-2+G2N1-2+G2]

G2L1-4: G2 RG4s with loop length between 1 and 4 nt [G2N1-4+G2N1-4+G2N1-4+G2]

G2L1-9: G2 RG4s with loop length between 1 and 9 nt [G2N1-9+G2N1-9+G2N1-9+G2]

In definitions, N represents A, C, G or U while H represents A, C or U.

The G-rich region sites identified with K^+^ or K^+^+PDS conditions were listed in Additional file 2: Table S1 and Table S2, respectively.

### Undetected G-rich regions

G-rich regions of *Arabidopsis* transcriptome were predicted by Quadbase 2 with non-overlapping and non-greedy settings. The predicted regions with an average coverage above 60 of Li^+^ channel in rG4-seq, not overlapped with the detected regions, and 50-nt flanking sequences were pooled as undetected G-rich regions.

### RNA secondary structure prediction

We used *RNAfold* from *ViennaRNA* (version 2.3.3) with options ‘-p -d2 -T 22.0’ for sequences folding at 22 °C and the base-pairing probability corresponding to MFE was extracted.

### Gini index and folding score calculation

Gin index was calculated from the SHALiPE-Seq libraries with reads number of G residues in G-tracts as described with modifications [17].

1 $$ \mathrm{Gini}=\frac{\sum_{i=1}^n{\sum}_{j=1}^n\mid ri- rj\mid }{2{n}^2\overline{r}} $$

*n* denotes the number of G residues in the G-tracts (continuous runs of guanine in the G-rich region), *ri* denotes the reads number in SHALiPE profiles at position *i*, and $\overline{r}$ denotes the average reads number of all G residues.

To calculate in vivo folding score of RG4s, the regions with Gini (in vitro K^+^)/Gini (in vitro Li^+^) ≥ 1.1 and G residues with average read count ≥ 50 reads/nt were included.

2 $$ \mathrm{folding}\ \mathrm{score}=\frac{\mathrm{Gini}\left(\mathrm{in}\ \mathrm{vivo}\right)-\mathrm{Gini}\left(\mathrm{in}\ \mathrm{vitro}\ {\mathrm{Li}}^{+}\right)}{\mathrm{Gini}\left(\mathrm{in}\ \mathrm{vitro}\ {\mathrm{K}}^{+}\right)-\mathrm{Gini}\left(\mathrm{in}\ \mathrm{vitro}\ {\mathrm{Li}}^{+}\right)} $$

### Gene Ontology analysis

Enrichment analysis of GO categories was performed online by the Gene Ontology Consortium (http://geneontology.org). The genes with G-rich regions identified by rG4-seq in the presence of K^+^ for Arabidopsis, or genes with average RT read counts ≥ 50 on predicted G-rich regions for rice, were used. The GO categories with a *P* value smaller than 0.05 were listed.

### Statistical hypothesis testing

All analysis was performed using the R programming language (http://www.r-project.org). Statistical methods and significance are indicated in the main text or figure legends for individual tests.

### Accession numbers

Sequence data have been deposited in the Sequence Read Archive (SRA) (https://www.ncbi.nlm.nih.gov/sra) under BioProject ID number PRJNA561194.

## Supplementary information

**Additional file 1: Figure S1.** rG4-seq libraries with high reproducibility. **Figure S2.** rG4-seq profiles and predicted secondary structure of the undetected G-rich region on *AT4G24820*. **Figure S3.** NAI probing of Arabidopsis 18S rRNA in vitro and in vivo, and high reproducible SHALiPE-Seq libraries. **Figure S4.** Comparison of Gini values of SHALiPE-seq on in vitro folded RG4s. **Figure S5.** Landscape of RG4s folded in vivo in *Arabidopsis*. **Figure S6.** Gene Ontology (GO) analysis reveals enrichment of genes with similar molecular functions in *Arabidopsis* and rice for the genes containing RG4s. **Figure S7.** RG4 on HIRD11 modulates plant growth and translation. **Figure S8.** Dual luciferase reporting assay reveals that the RG4 on the 3’UTR of *HIRD11* regulates translation.

**Additional file 2: Table S1.** G-rich regions with folding potential identified by rG4-seq with K^+^. **TableS2.** G-rich regions with folding potential identified by rG4-seq with K^+^+PDS. **TableS3.** Gini index of SHALiPE profiles in vitro in the presence of Li^+^ and K^+^. **TableS4.** In vivo folding scores of Arabidopsis G-rich regions. **TableS5.** In vivo folding scores of rice G-rich regions. **TableS6.** Gene pairs of orthologues with RG4s in *Arabidopsis* and rice. **TableS7.** Primers used in this study.

**Additional file 3.** Review history.

## Data Availability

Sequence data have been deposited in the Sequence Read Archive (SRA) (https://www.ncbi.nlm.nih.gov/sra) under BioProject ID number PRJNA561194 [58]. Ribosome profiling data are available in the National Center for Biotechnology Information’s Gene Expression Omnibus under accession number GSE43703 [35].
