# Supplementary material for: RNA G-quadruplex structures exist and function in vivo in plants
Source: Genome Biol. 2020 Sep 1;21:226. doi: 10.1186/s13059-020-02142-9 (PMC7466424; doi:10.1186/s13059-020-02142-9)
Supplement: Supplementary file 1 — Additional file 1: Figure S1. rG4-seq libraries with high reproducibility. Figure S2. rG4-seq profiles and predicted secondary structure of the undetected G-rich region on AT4G24820. Figure S3. NAI probing of Arabidopsis 18S rRNA in vitro and in vivo, and high reproducible SHALiPE-Seq libraries. Figure S4. Comparison of Gini values of SHALiPE-seq on in vitro folded RG4s. Figure S5. Landscape of RG4s folded in vivo in Arabidopsis. Figure S6. Gene Ontology (GO) analysis reveals enrichment of genes with similar molecular functions in Arabidopsis and rice for the genes containing RG4s. Figure S7. RG4 on HIRD11 modulates plant growth and translation. Figure S8. Dual luciferase reporting assay reveals that the RG4 on the 3’UTR of HIRD11 regulates translation. [file 13059_2020_2142_MOESM1_ESM.docx]

**Figure S1**


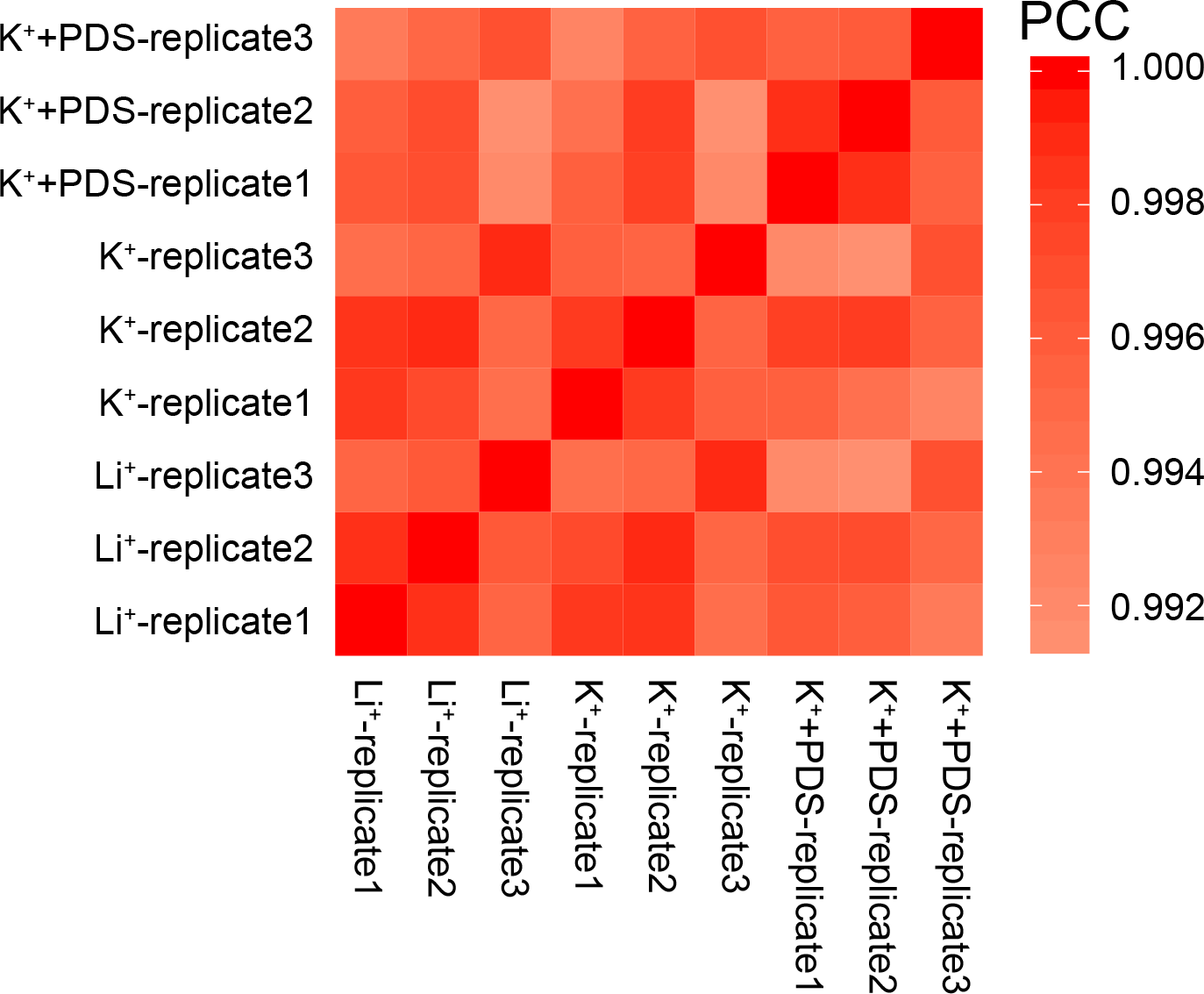


**Figure S1** rG4-seq libraries with high reproducibility

High correlations of mRNA expression levels among biological replicates of rG4-seq libraries.

**Figure S2**


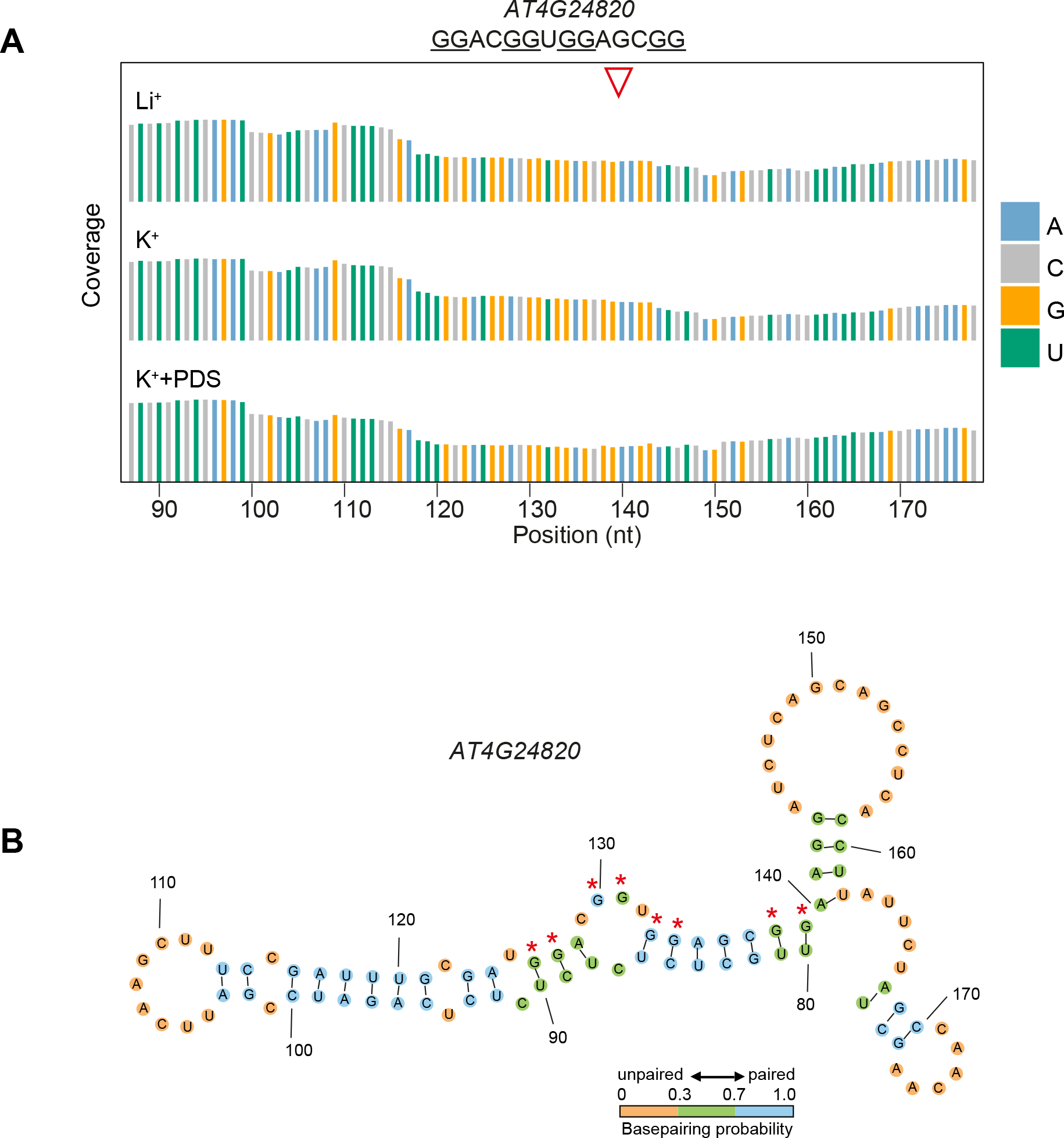


**Figure S2** rG4-seq profiles and predicted secondary structure of the undetected G-rich region on *AT4G24820*

**(A)** rG4-seq profiles of the predicted and undetected G-rich region on *AT4G24820,* displayed the reads coverage of reverse transcription (RT) with Li^+^ (top), K^+^ (middle), and K^+^+PDS (bottom) respectively. The 3’end of the G-rich region site was indicated by red coloured triangle. A (blue), C (light grey), G(yellow), U(green).

**(B)** Secondary structure of G-rich region and flanking sequences on *AT4G24820*, predicted using Vienna RNA fold. The filling colours of orange, green and blue indicate the base pairing probability of below 0.3, 0.3-0.7 and above 0.7 respectively. Red stars indicate the guanines consisting the predicted G-rich region, which are strongly base paired with flanking sequences. Numbers indicate positions of the nucleotides on the transcript.

**Figure S3**


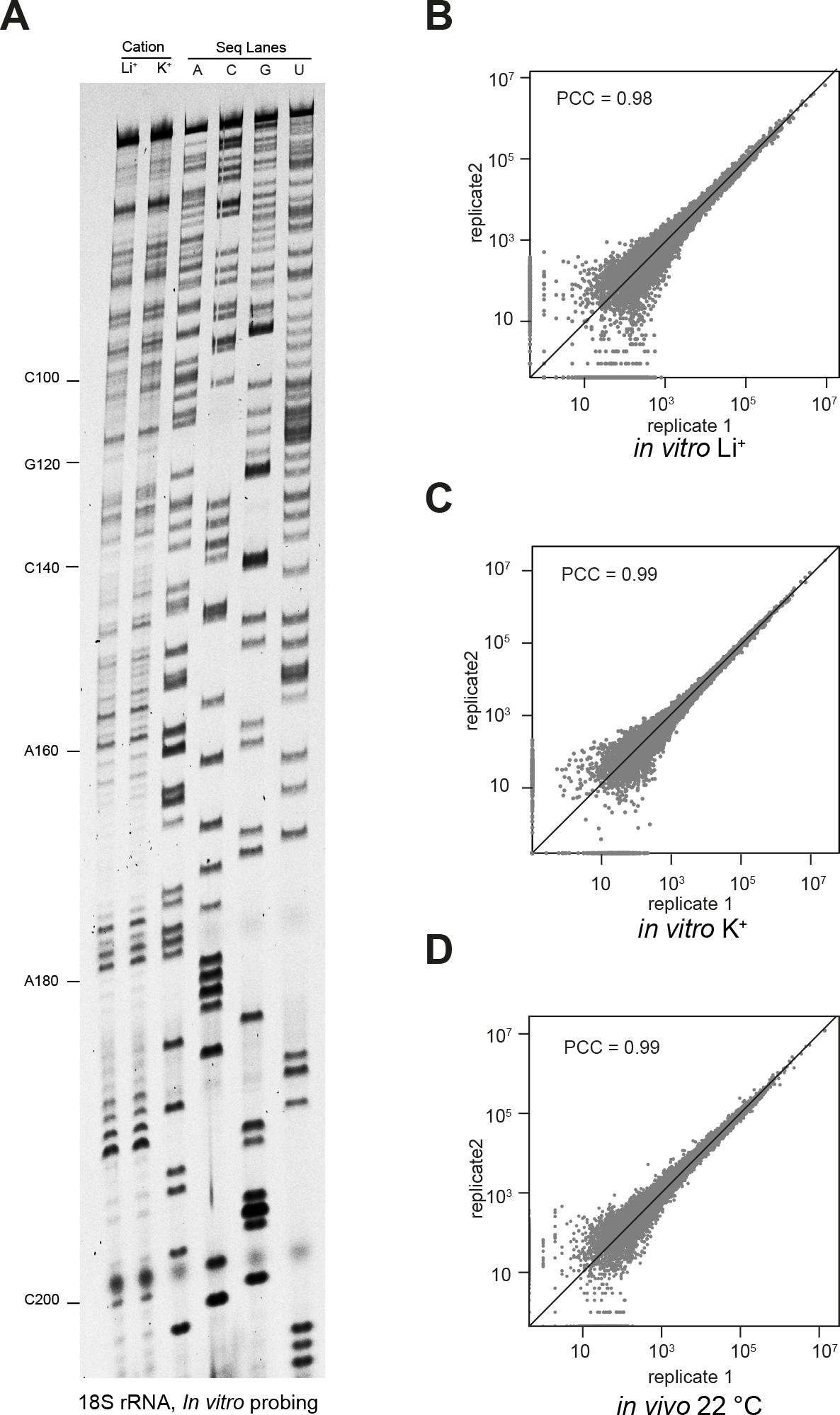


**Figure S3** NAI probing of *Arabidopsis* 18S rRNA *in vitro* and *in vivo*, and high reproducible SHALiPE-Seq libraries

**(A)** Comparable modification of NAI probing *in vitro* with Li^+^ or K^+^, illustrated by the fragment of *Arabidopsis* 18S rRNA with gel-based analysis.

**(B)-(D)** High correlations of mRNA expression levels between two independent biological replicates of the SHALiPE-Seq libraries for *in vitro* with Li^+^ (B), *in vitro* with K^+^ (C) and *in vivo* at 22°C (D).

**Figure S4**


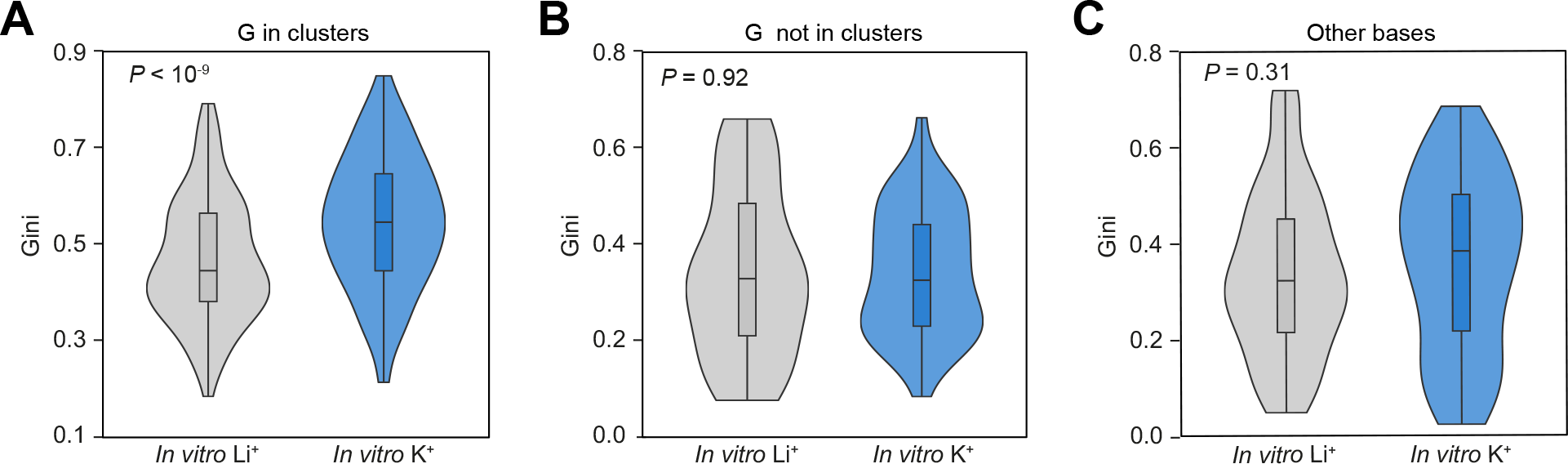


**Figure S4** Comparison of Gini values of SHALiPE-seq on *in vitro* folded RG4s.

**(A)** Violin plot showing Gini *in vitro* with Li^+^ and Gini *in vitro* with K^+^ on the Gs in clusters. Gini with K^+^ is significantly higher than Gini with Li^+^ (*P* < 10^-9^). Regions with average counts ≥ 50 on the bases were included for calculation. *P*-value, paired Student’s t-test.

**(B)** Violin plot showing Gini *in vitro* with Li^+^ and Gini *in vitro* with K^+^ on the Gs not in cluster. Gini with K^+^ is similar to Gini with Li^+^ (*P* = 0.92). Otherwise in Figure S4A.

**(C)** Violin plot showing Gini *in vitro* with Li^+^ and Gini *in vitro* with K^+^ on the other bases (A, C and U). Gini with K^+^ is similar to Gini with Li^+^ (*P* = 0.31). Otherwise in Figure S4A.

**Figure S5**


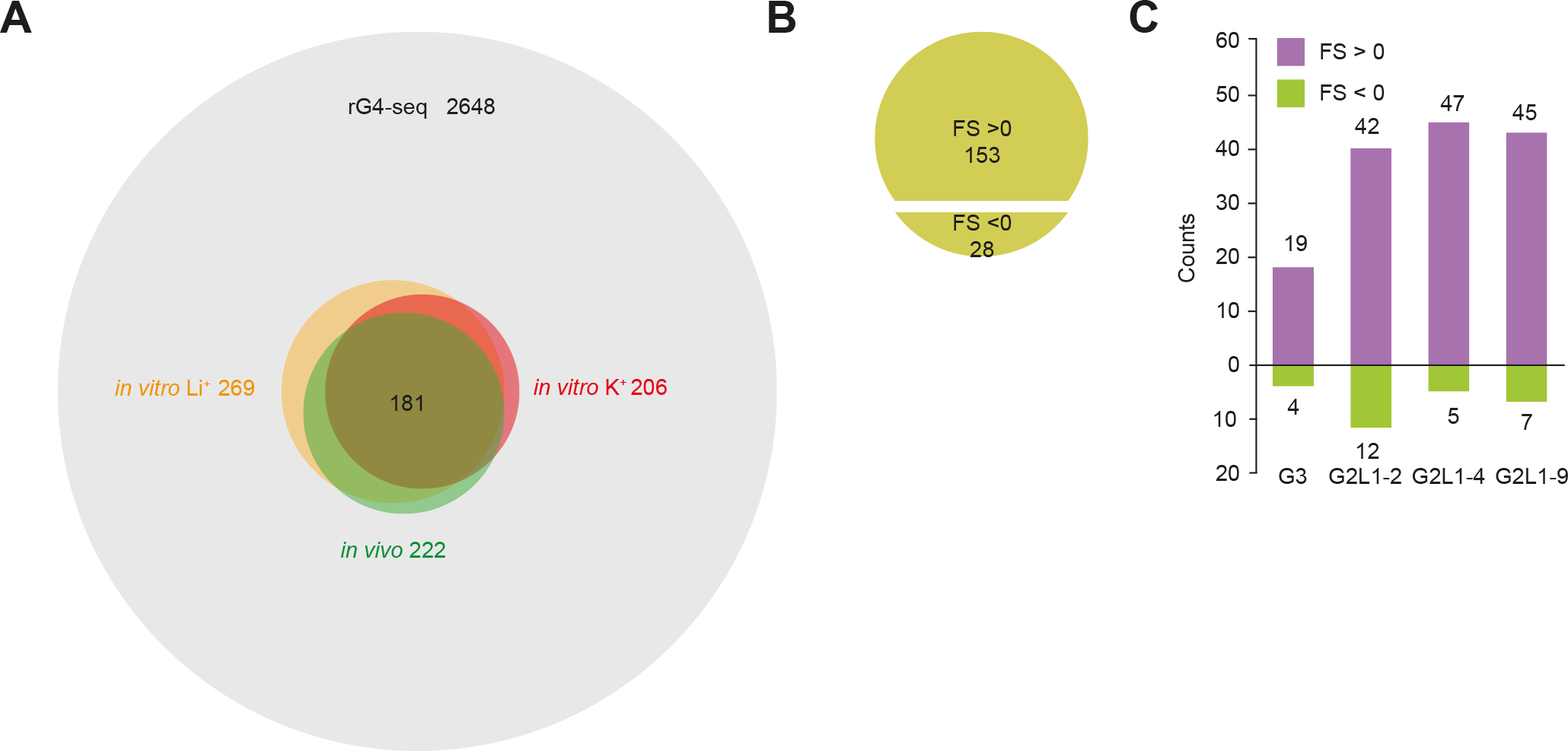


**Figure S5** Landscape of RG4s folded *in vivo* in *Arabidopsis*

**(A)** Venn diagram showing the number of regions for analysis in each dataset. rG4-seq identified 2648 G-rich regions folding into RG4 *in vitro*. Using a cut-off of Gini (*in vitro* K^+^) / Gini(*in vitro* Li^+^) ≥ 1.1 and average reads count ≥ 50 on guanines, 269, 206 and 222 regions were found in SHALiPE-seq libraries for the conditions of *in vitro* with Li^+^, *in vitro* with K^+^ and *in vivo* respectively, with an overlap of 181 regions detected. These regions were further subjected to calculating *in vivo* folding scores.

**(B)** Among the 181 regions in *Arabidopsis,* 153 regions with *in vivo* folding score (FS) > 0 and 28 regions with FS < 0.

**(C)** Counts of the regions with folding score > 0 or folding score < 0 for different types of RG4s.

**Figure S6**


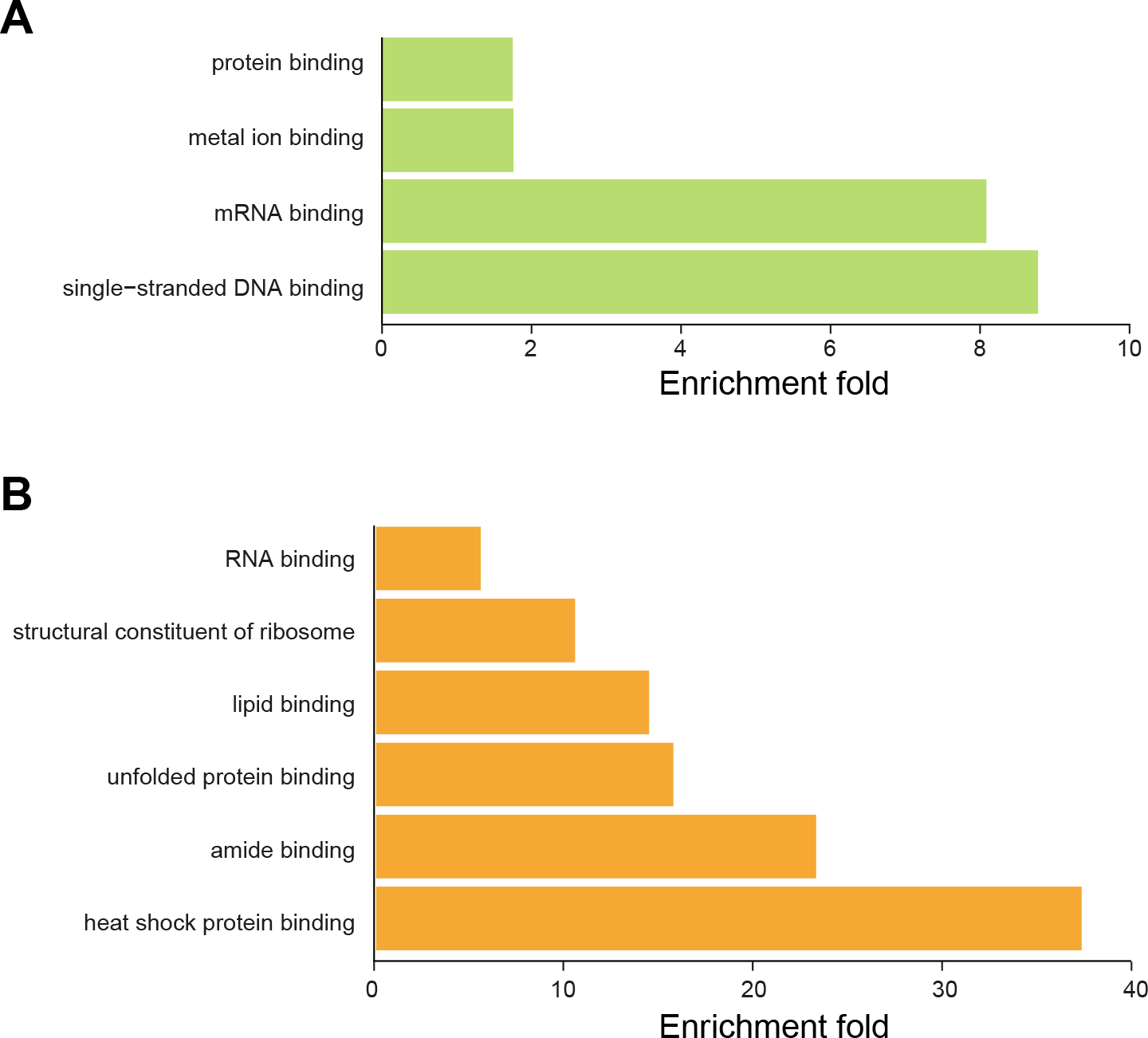


**Figure S6** Gene Ontology (GO) analysis reveals enrichment of genes with similar molecular functions in *Arabidopsis* and rice for the genes containing RG4s.

**(A)** GO terms of molecular functions enriched for the genes with RG4s in *Arabidopsis* (*P* < 0.05) were listed. *P*-value, Fisher’s exact-test with Bonferroni correction.

**(B)** GO terms of molecular functions enriched for the genes with RG4s in rice (*P* < 0.05) were listed. Otherwise in Figure S6A.

**Figure S7**


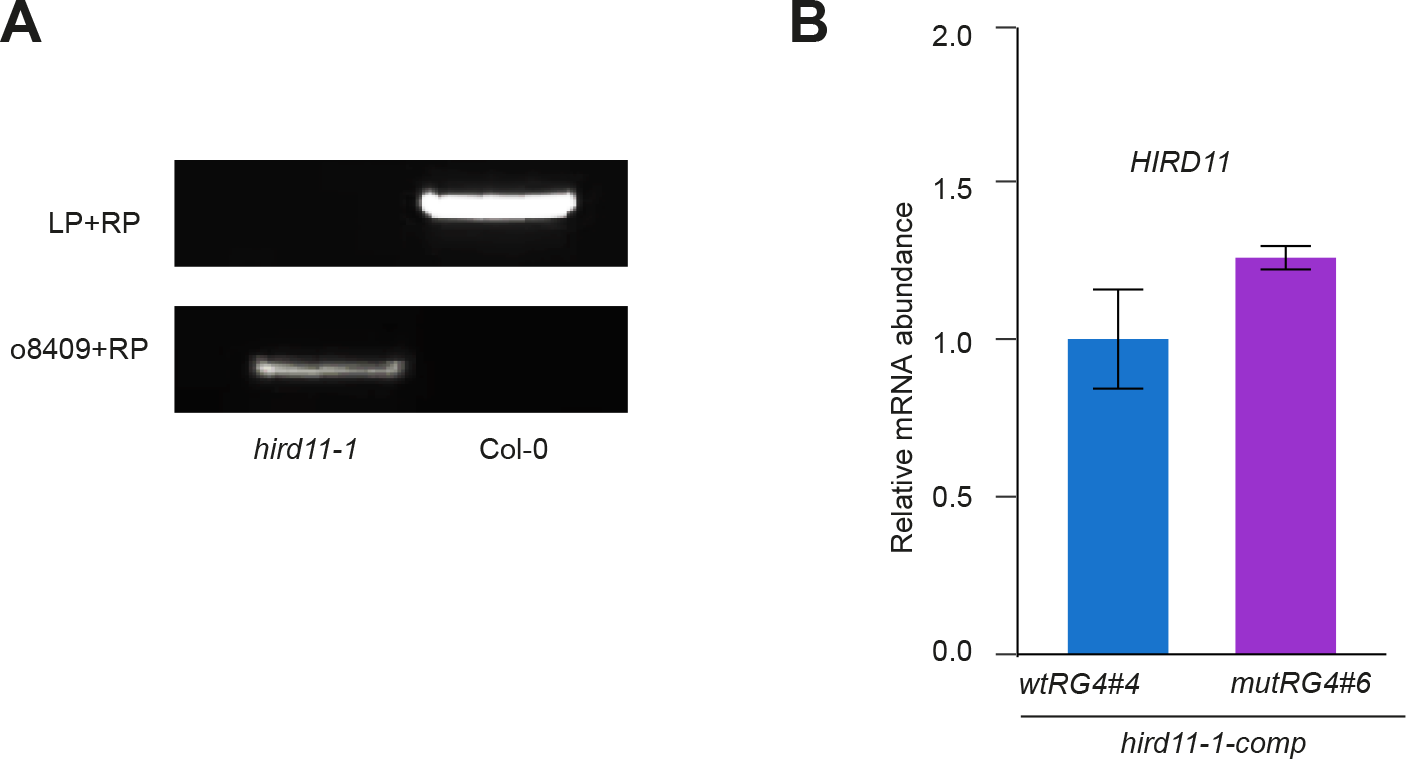


**Figure S7** RG4 on *HIRD11* modulates plant growth and translation

**(A)** PCR based genotyping of the *hird11-1* mutant. PCR was conducted with genomic DNA from *hird11-1* using a pair of *HIRD11*-specific primers (LP+RP) or by the combination of T-DNA left border–specific primers (o8409) and a corresponding *HIRD11*–specific primer (RP). PCR was parallelly performed with genomic DNA from Col-0 plants.

**(B)** Relative mRNA abundance of *HIRD11* in *hird11-1-comp-wtRG4* and *hird11-1-comp-mutRG4* plants. mRNA abundance was similar between wtRG4 and mutRG4 plants, *P* = 0.231, Student’s t-test, error bars indicate SE.

**Figure S8**

**
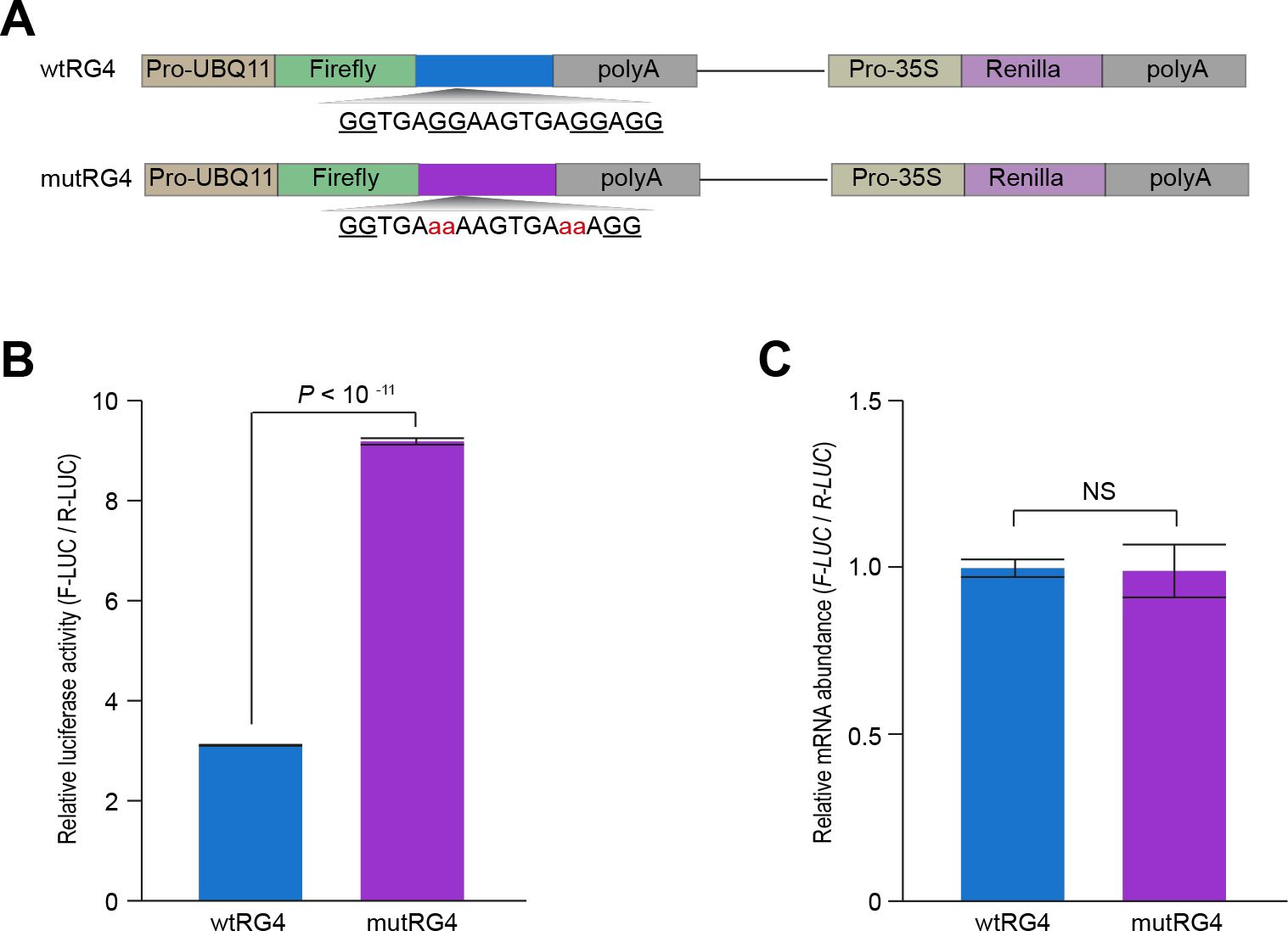
**

**Figure S8** Dual luciferase reporting assay reveals that the RG4 on the 3’UTR of *HIRD11* regulates translation

**(A)** Schematic diagram of vectors employed in dual luciferase reporting assay. *HIRD11* 3’UTR containing wtRG4 and mutRG4 were fused with the coding region of Firefly luciferase, respectively.

**(B)** Comparison of Firefly luciferase (F-LUC) activity of tobacco leaves expressing the Firefly luciferase gene fused with the 3’UTR of *Arabidopsis HIRD11* with wtRG4 or mutRG4, respectively. Firefly luciferase was normalized to the internal control, Renilla luciferase (R-LUC). The luciferase activities of 8 independent biological replicates were measured. *P*-value, Student’s t-test, error bars indicate SE.

**(C)** mRNA abundance of *F-LUC* of tobacco leaves expressing the Firefly luciferase gene fused with the 3’UTR of *Arabidopsis HIRD11* with wtRG4 or mutRG4, respectively. Abundance of *F-LUC* mRNA was normalized to *R-LUC* mRNA abundance. Otherwise in Figure S8B.
