# Supplementary material for: RNA G-quadruplex structures exist and function in vivo in plants
Source: Genome Biol. 2020 Sep 1;21:226. doi: 10.1186/s13059-020-02142-9 (PMC7466424; doi:10.1186/s13059-020-02142-9)
Supplement: Supplementary file 3 — Additional file 3. Review history. [file 13059_2020_2142_MOESM3_ESM.docx]

Review History

**First round of review**

**Reviewer 1**

**Are you able to assess all statistics in the manuscript, including the appropriateness of statistical tests used?**

No, I do not feel adequately qualified to assess the statistics.

**Comments to author:**

In this manuscript Ding and colleagues demonstrate the existence of RNA G-quadruplex structures in living plants, which is in stark contrast to what reported for yeast and mESCs using DMS-based approaches. The paper is extremely well-written and is based on a strong RG4-seq in vitro mapping that enables comparisons between the RG4 landscape observed in vitro to what observed in vivo. Undoubtedly this manuscript should be considered for publication in Genome Biology given the quality of the study and the general impact of the findings that support, for the first time, RNA G4 formation in a living organism.

However there are a couple of major concerns that the authors should address prior publication:

1) The weakest component of this study is the investigation of the biological functions of RG4 in plants, where the authors speculate that their formation on 5 UTR site stimulate translation. Whilst the authors provide compelling evidence to demonstrate that this is the case for HIRD11, I feel that this is not a statement that can be generalised based on this evidence and this is not reflected on the title of the section (RG4 structure regulates translation and plant development), which instead sounds rather generic and a bit misleading. I would suggests to the authors to see if inserting the RG4 present on the 5 UTR of HIRD11 has similar effects in terms of translation stimulation also to other mRNA that might contain no G4 at all or, alternatively, extend this analysis to other 5 UTR that contain RG4s. Without this evidence is hard to have a clear correlation between RG4 formation and translation efficiency, as mutation of this particular 5' UTR might have a detrimental effect on the translational level on his own right independently from G4-formation. If the authors are not in a position to perform experiments given the current COVID-19 situation, I feel that the manuscript is sufficiently interesting and important without speculation on the specific biological role that these RG4s might have and this part might be omitted (or significantly re-written) to avoid making misleading speculations and jump to conclusions not fully supported by data.

2) What is the effect of using SHALIPE vs DMS in terms of their final observations? The authors seem to suggest that RG4s are detected in plants are owing to the different nature of the species observed (compared to yeast and mESCs), but they use a different technique to map RG4s in vivo. A caveat on what could be the effect of changing technique also in the light of previous report should be added. The authors mention that high concentrations of DMS might degrade RNA, couldn't this be happening also in yeast and mESCs?

Minor points:

3) The is no discussion on how the RG4-seq maps (of ~6000 G4s detected) compares to what is observed in vivo, the authors mention only hundreds of RG4s detected in vivo, it would be valuable to discuss which ones are actually formed among the ~6000 detected in vitro. Does the landscape of in vivo detected RG4s change during plant development?

**Reviewer 2**

**Are you able to assess all statistics in the manuscript, including the appropriateness of statistical tests used?**

No, I do not feel adequately qualified to assess the statistics.

**Comments to author:**

In this manuscript, the authors report the existence of RNA G-quadruplex (RG4) structures in Arabidopsis and rice. This is the first study showing the in vivo existence of RG4s in eukaryotes (previous studies concluded on the absence of such structures in yeast or human cells in vivo). The authors hypothesized that RG4 structures might form in vivo in plants as plant cells have higher concentrations of K+, which induces RG4 formation in vitro. They first identified G-rich regions that can form RG4s in vitro through rG4-seq, and showed that only a very small fraction of G-rich regions form RG4s in vitro. They showed that the G-rich regions that do not form RG4s are predicted to form secondary structures, suggesting that secondary structures inhibit RG4 formation. They next performed SHALiPE-seq to map RG4s in vivo in both Arabidopsis and rice and found that regions that form RG4s in vitro indeed have high folding scores in vivo. They further examined the biological role of one RG4 in the 3' UTR of the Arabidopsis gene HIRD11. Mutating multiple Gs to As in the region disrupted the ability of the gene to rescue the mutant phenotype and reduced the polysome association of the mRNA.

Overall, this is a well-performed study that revealed the existence of RG4s in eukaryotes for the first time. The work should be of interest to the RNA biology community beyond the plant field and opens new avenues of research.

This reviewer feels that the authors can make better uses of their genomics data to provide more information to the community. As of now, the data analyses are rather limited in scope.

1. In the SHALiPE-seq analysis, the authors only focused on the G-rich regions that were shown to form RG4s in vitro (maybe I am wrong about this, if so, at least the authors need to be clearer about what G-rich regions were analyzed). With the in vivo data, it is easy to examine all G-rich regions that are predicted to form RG4s.
2. In fact, related to 1, it would be interesting to know whether the same regions form RG4 in vivo vs. in vitro. Of course, for this, one has to be able to identify RG4s through SHALiPE-seq. The authors did not call RG4s with SHALiPE-seq. Are there technical difficulties for this?
3. Related to 1 and 2, is SHALiPE-seq specific for RG4 detection? For Gs that are not in clusters, do you see differences in Gini? How about other nucleotides? This reviewer wonders whether other features of RNA, such as RNA modification or secondary structure, confound the interpretation of SHALiPE-seq for RG4.
4. It would be nice to know whether the same genes tend to form RG4s in their RNAs in Arabidopsis and rice. Are there specifically enriched GO terms?
5. The authors concluded that G-rich regions that do not form RG4s in vitro are more likely to form secondary structures. This was based on RNA structure prediction. But the authors actually have profiled RNA structures in vivo. Why not confirm their prediction-based conclusion with available information on RNA secondary structures in vivo? They have both in vivo RG4 and in vivo secondary structure information.

In addition to the comments above on the bioinformatics analyses, there are concerns on the functional study of the RG4 in HIRD11. Please mention how many independent transgenic lines were evaluated for the rescue experiment. Also, the mutations not only abolish RG4 formation but also potentially change the secondary structure and definitely change the sequence of the RNA, thus the defects associated with the mutant transgene do not necessarily reflect the function of the RG4. It is hard to get around this issue, but at least the authors need to discuss this point.

**Reviewer 3**

**Are you able to assess all statistics in the manuscript, including the appropriateness of statistical tests used?**

Yes, and I have assessed the statistics in my report.

**Comments to author:**

Although G-rich sequences are abundant in eukaryotic cells, little is known the existence of RNA G-quadruplex structure in vivo in plants and how it can control translation and plant development. In this study, Yang et al. determined in vitro (K+, PDS) and in vivo (2-methylnicotinic acid imidazolide (NAI)) RNA chemical structure profiling to determine whether RG4 structures are folded in Arabidopsis and rice. The authors explicitly provided the evidence of RG4 folding formation in vivo via SHALiPE-seq in both plants. They also proved the physiological role of RG4 in vivo by showing that RG4 inhibits translation of HIRD11 gene as an example in Arabidopsis which controls plant root growth. Conclusively, this manuscript illustrates the existence of folded RG4in vivo and emphasizes the functional role of RG4 in plants. However, regardless of the novelty of this study, there are several drawbacks that might hamper to understand the functional roles of RG4s in various aspects of plant growth and development.

1. In Figure 1h, it would be informative to make a plot of showing a proportional distribution of RG4s across genes by plotting RG4s in the start-end of all genes with RG4s in 2kb-window. That is, where are RG4s exactly enriched in plant gene structures? Gene ends or the middle of genes or uniform across gene? It might be necessary to compare the sequenced RG4 with predicted in silico RG4. Why not showing the same graph or plot for in vivo SHALiPE profiles in Figure 3-5? Please consider to add gene structure on rG4seq or SHALiPE seqs (in vivo, in vitro +K+ and Li+) in Figure 1-5, which will show the location of RG4s in gene structure (5´UTR, gene body, 3´UTR).

2. It is shown that RG4s are enriched at CDS in plants. Do these gene body RG4s also affect their corresponding translation? if so, what would be a mechanistic base of gene body-seeded translational regulation? The authors claimed that the location of RG4s in gene doesn't affect RG4 structure folding, but there is no evidence showing how CDS RG4s affect translation in plants, unlike the cases that SMXL5 has RG4 at 5´UTR and HIRD11 at 3´UTR for translation inhibition.

3. In respect to in vivo functional role of RG4s in translation, it would be necessary to show ribosome-seq profile patterns of wild-type plants at the same plot of RG4 distribution in all genes with RG4s and all genes without RG4s, together with RNA seq profiles if necessary.

4. It would be informative if Venn diagrams are presented showing how many genes with RG4s are overlapped and separated between rG4seq or SHALiPE seqs (in vivo, in vitro +K+ and Li+) in Arabidopsis and rice. It would be intriguing if there is any preference of RG4 structures on in vitro, in vivo, or both, which I think give us a hint for prediction and properties of RG4 structure formation.

5. The authors examined the 5´UTR of SMXL5 to validate rG4seq in Figure 1. Why not showing the representative locus for SHALiPE profiles (in vivo, in vitro +K+ and Li+)? If not, is there any reason?

6. In Figure 5 and Fig S4, mutated RG4 HIRD11 transcripts were shown to be enriched in polysome fraction and no change in mRNA level. Is there any change in protein level?

7. Given that functional RG4 structures, which are known to be globally unfolded in mammalian and yeast cells, exist in Arabidopsis and rice, I wonder whether RG4 structures are conserved in plants. To this end, it might be helpful to analyze sequence homology of genes with RG4 structure in Arabidopsis and rice.

Minor comments;
1. In page 10 line 14, 'presence of K+ (0.67 and 0.52)' should be 'presence of K+ (0.67 and 0.56)'.
2. In the method part for alignment of the sequencing reads, it would be helpful to provide parameters for bowtie alignment and handling process of aligned read such as filtering of mapping quality, how multiple mapped reads were processed.
3. In Gini index, page 26 line 27 for 'G residues in the G-tracts', what does the G-tracts present? Dose it mean G-rich regions? How G-tracts were defined?
4. In Gini index, what γ¯ does stand for the mean of reads number of γi ?

**Authors Response**

**Point-by-point responses to the reviewers’ comments:**

Comment

*Response*

**Reviewer #1**

In this manuscript Ding and colleagues demonstrate the existence of RNA G-quadruplex structures in living plants, which is in stark contrast to what reported for yeast and mESCs using DMS-based approaches. The paper is extremely well-written and is based on a strong RG4-seq in vitro mapping that enables comparisons between the RG4 landscape observed in vitro to what observed in vivo. Undoubtedly this manuscript should be considered for publication in Genome Biology given the quality of the study and the general impact of the findings that support, for the first time, RNA G4 formation in a living organism. However there are a couple of major concerns that the authors should address prior publication:

*RESPONSE*

*We thank the Referee for their appreciation that our paper:“is extremely well-written and is based on a strong RG4-seq in vitro mapping that enables comparisons between the RG4 landscape observed in vitro to what observed in vivo. Undoubtedly this manuscript should be considered for publication in Genome Biology given the quality of the study and the general impact of the findings that support, for the first time, RNA G4 formation in a living organism.” We are very appreciative of the time and effort that the Referee has spent assessing our work and for their numerous suggested improvements. We have edited and improved our manuscript accordingly.*

Comment 1

The weakest component of this study is the investigation of the biological functions of RG4 in plants, where the authors speculate that their formation on 5 UTR site stimulate translation. Whilst the authors provide compelling evidence to demonstrate that this is the case for HIRD11, I feel that this is not a statement that can be generalised based on this evidence and this is not reflected on the title of the section (RG4 structure regulates translation and plant development), which instead sounds rather generic and a bit misleading. I would suggests to the authors to see if inserting the RG4 present on the 5 UTR of HIRD11 has similar effects in terms of translation stimulation also to other mRNA that might contain no G4 at all or, alternatively, extend this analysis to other 5 UTR that contain RG4s. Without this evidence is hard to have a clear correlation between RG4 formation and translation efficiency, as mutation of this particular 5' UTR might have a detrimental effect on the translational level on his own right independently from G4-formation. If the authors are not in a position to perform experiments given the current COVID-19 situation, I feel that the manuscript is sufficiently interesting and important without speculation on the specific biological role that these RG4s might have and this part might be omitted (or significantly re-written) to avoid making misleading speculations and jump to conclusions not fully supported by data.

*RESPONSE*

*We are very grateful to the Referee for their suggestions and kind consideration. We have followed the reviewer’s suggestion and performed dual luciferase reporting assay as follows. 3’UTR of HIRD11 containing wildtype RG4 sequence (wtRG4) or mutated RG4 sequence (mutRG4) was fused with the Firefly luciferase reporter gene, with Renilla luciferase used as the internal control. The luciferase activities of 8 independent biological replicates were measured. We found that the Firefly luciferase activity (F-LUC) of mutRG4 was ~3 times higher to that of wtRG4, when normalized to the Renilla luciferase activity (R-LUC) (Rebuttal Figure 1A, P < 10 -11, Student’s t-test). We also measured RNA abundance and found no significant differences between wtRG4 and mutRG4 (Rebuttal Figure 1B). These new results are shown in the new Figure S8 and described from line 278 to line 284 on page 13 in the revised manuscript.*

*In addition, we calculated the translation efficiency of transcripts containing either detected RG4s or undetected RG4s using the ribosome profiling data in Arabidopsis [1]. We found that translation efficiency (TE) values of those mRNAs containing folded RG4s are significantly lower than those without folded RG4s (Rebuttal Figure 2, new Fig. 5a). Therefore, from the results on individual examples of HIRD11 and global analysis of ribosome profiling, we conclude that folded RG4s tend to have a general effect of repressing translation efficiency of mRNAs. We have added the results in the lines 249 to 255 on page 12 in the revised manuscript.*

*Rebuttal Figure 1 Dual luciferase reporting assay reveals that the RG4 on the 3’UTR of HIRD11 regulates translation*

*(A) Comparison of Firefly luciferase (F-LUC) activity of tobacco leaves expressing the Firefly luciferase gene fused with the 3’UTR of Arabidopsis HIRD11 with wtRG4 or mutRG4, respectively. Firefly luciferase was normalized to the internal control, Renilla luciferase (R-LUC). The luciferase activities of 8 independent biological replicates were measured. P-value, Student’s t-test, error bars indicate SE.*

*(B) mRNA abundance of F-LUC of tobacco leaves expressing the Firefly luciferase gene fused with the 3’UTR of Arabidopsis HIRD11 with wtRG4 or mutRG4, respectively. Abundance of F-LUC mRNA was normalized to R-LUC mRNA abundance. Otherwise in Rebuttal Figure 1A.*

*Rebuttal Figure 2 RG4 regulates translation*

*Violin plot of translation efficiency (TE) of transcripts for predicted but undetected RG4s (named as RG4 undetected) and detected RG4s (named as RG4 detected). TE of transcripts with detected RG4s is significantly lower than that of RG4 undetected transcripts (P < 10-13, Student’s t-test).*

Comment 2 What is the effect of using SHALIPE vs DMS in terms of their final observations? The authors seem to suggest that RG4s are detected in plants are owing to the different nature of the species observed (compared to yeast and mESCs), but they use a different technique to map RG4s in vivo. A caveat on what could be the effect of changing technique also in the light of previous report should be added. The authors mention that high concentrations of DMS might degrade RNA, couldn't this be happening also in yeast and mESCs?

*RESPONSE*

*We thank the Referee for these insightful comments. We originally developed the SHALiPE method for individual in vitro RG4 detection[2]. Here, we extended our SHALiPE method for detecting RG4s at the genome wide scale. Notably, we found that RNA G-quadruplex structures are globally folded in plants by applying the SHALiPE method in both Arabidopsis and rice. Generally, SHALiPE is robust in determining the folding status of G-rich regions in vitro and in vivo at both individual and genome-wide scale studies.*

*Although both SHALiPE and DMS methods have been applied to studying RG4 folding, they are quite different to each other. The SHALiPE method, utilising specific SHAPE modification, searches for “positive” signals directly on the folded RG4s. Whereas the basis for the DMS method is that a high dose of DMS has modified all N7G sites when the G is not involved in RG4 formation, i.e. the “free site” of the hoogsteen base-pairing face of the guanines. In other words, the DMS method searches for “negative” signals in detecting RG4 folding status. Additionally, we have also discussed the concerns about the DMS over-methylation which might lead to unpredictable inaccuracies of RG4 structure determination in our previous review paper[3]. In contrast, the SHALiPE method is performed under single-hit kinetics conditions. Moreover, the Guo et al., study used over 8% of DMS for yeast and mESCs. In plants, 2% and 5% DMS were reported to cause browning of plants, which might be due to the reaction of DMS with chlorophyll molecules[4]. 2% DMS also can cause visible RNA decay although the precise mechanism is not clear[4]. Due to the relatively low tolerance of DMS in plants compared to yeast, it is not possible to achieve the over-modification level of all N7G sites by DMS alone. We have edited the corresponding content in the lines 161-164 by adding new references in the revised manuscript.*

Minor points:

Comment 3 There is no discussion on how the RG4-seq maps (of ~6000 G4s detected) compares to what is observed in vivo, the authors mention only hundreds of RG4s detected in vivo, it would be valuable to discuss which ones are actually formed among the ~6000 detected in vitro. Does the landscape of in vivo detected RG4s change during plant development?

*RESPONSE*

*We thank the Referee for this comment.*

*Using rG4-seq under the conditions of both K+ and K++PDS in Arabidopsis, 2648 regions were detected in total. We then used SHALiPE to further confirm the in vitro detected RG4s from rG4-seq. Since we selected those RG4 sites with a cut-off of an average RT stop reads count ≥ 50 on guanines in all three SHALiPE-seq libraries (in vitro Li+ and in vitro K+ and in vivo) along with the threshold of Gini K+/ Gini Li+ ≥ 1.1 (in vitro folded sites), this step resulted in an overlap of 181 RG4 sites (Rebuttal Figure 3A). By calculating the in vivo folding score with in vivo Gini, we found 153 regions with folding scores over 0 (84% >0), indicating that the folding of these RNA G-quadruplex structures (84%) are detectable in living plants, while only 28 regions have folding scores lower than 0 (Rebuttal Figure 3B). Therefore, most RG4s sites folded in vitro tended to be folded in vivo as well. Following the Referee’s excellent point, we also plotted the detailed numbers of different types of RG4s (Rebuttal Figure 3C). We found that most of RG4s with different types folded in vitro tend to form in vivo in Arabidopsis (Rebuttal Figure 3C). However, there are slightly more G2 RG4s folded in vivo having longer loop lengths (G2L1-4 and G2L1-9), as compared to G2L1-2. These results imply that those G2 RG4 with longer loop lengths may be more strongly folded in vivo, which will be interested in the further investigation.*

*Since we used a cut-off of very high sequencing depth (average reads ≥ 50) to confidently estimate the folding of RG4s, the number of RG4 sites detected in vivo is quite smaller than the 2648 RG4 sites detected by rG4-seq. Therefore, it is possible that there may be more in vivo folded RG4s than the number we reported, which may warrant further investigation in the future. We have edited the discussion in our revised manuscript (line 327 to line 335 on page 16), accordingly.*

*A previous biophysics study suggested that the GQS structure tends to be more stable than the stem-loop structure at ~22 °C[5]. The whole process of plant development is mainly under ~22°C. It is likely that the global folding status of RG4s might remain relatively stable during plant development. However, it will be a very interesting future plant development study to investigate whether changes occur in the folding status of individual RG4s for those plant development-related mRNAs changes.*

*Rebuttal Figure 3 Structural landscape of folded RNA G-quadruplex features in Arabidopsis*

*(A) Venn diagram showing the number of regions for analysis in each dataset. rG4-seq identified 2648 G-rich regions folding into RG4 structure in vitro. 269, 206 and 222 regions with average reads count on guanines over 50 and Gini K+/ Gini Li+ ≥ 1.1 were found in SHALiPE-seq libraries for the conditions of in vitro with Li+, in vitro with K+ and in vivo respectively, resulting in an overlap of 181 regions. We focused on these 181 regions to calculate in vivo folding scores.*

*(B) In vivo folding score (FS) on the 181 regions in Arabidopsis, with folding score > 0 on 153 regions while folding score < 0 on the other 28 regions.*

*(C) Counts of regions with folding score > 0 or folding score < 0 of different types of RG4s.*

Reviewer #2:

Review of GBIO-D-20-00437

In this manuscript, the authors report the existence of RNA G-quadruplex (RG4) structures in Arabidopsis and rice. This is the first study showing the in vivo existence of RG4s in eukaryotes (previous studies concluded on the absence of such structures in yeast or human cells in vivo). The authors hypothesized that RG4 structures might form in vivo in plants as plant cells have higher concentrations of K+, which induces RG4 formation in vitro. They first identified G-rich regions that can form RG4s in vitro through rG4-seq, and showed that only a very small fraction of G-rich regions form RG4s in vitro. They showed that the G-rich regions that do not form RG4s are predicted to form secondary structures, suggesting that secondary structures inhibit RG4 formation. They next performed SHALiPE-seq to map RG4s in vivo in both Arabidopsis and rice and found that regions that form RG4s in vitro indeed have high folding scores in vivo. They further examined the biological role of
one RG4 in the 3' UTR of the Arabidopsis gene HIRD11. Mutating multiple Gs to As in the region disrupted the ability of the gene to rescue the mutant phenotype and reduced the polysome association of the mRNA.

Overall, this is a well-performed study that revealed the existence of RG4s in eukaryotes for the first time. The work should be of interest to the RNA biology community beyond the plant field and opens new avenues of research.

This reviewer feels that the authors can make better uses of their genomics data to provide more information to the community. As of now, the data analyses are rather limited in scope.

*RESPONSE*

*We thank the Referee for their appreciation that our work is: “a well-performed study that revealed the existence of RG4s in eukaryotes for the first time. The work should be of interest to the RNA biology community beyond the plant field and opens new avenues of research”. We are grateful for the time and effort that the Referee has spent in helping us strengthen our manuscript. Following the Referee’s suggestions, we have performed extensive new analyses to provide more information to the community.*

Comment 1 In the SHALiPE-seq analysis, the authors only focused on the G-rich regions that were shown to form RG4s in vitro (maybe I am wrong about this, if so, at least the authors need to be clearer about what G-rich regions were analyzed). With the in vivo data, it is easy to examine all G-rich regions that are predicted to form RG4s.

Comment 2 In fact, related to 1, it would be interesting to know whether the same regions form RG4 in vivo vs. in vitro. Of course, for this, one has to be able to identify RG4s through SHALiPE-seq. The authors did not call RG4s with SHALiPE-seq. Are there technical difficulties for this?

*RESPONSE*

*We thank the Referee for these two comments. In our study, we specifically aimed to explore the existence of RG4 in vivo, to resolve an important question which has been debated for decades. Therefore, it is critical to firstly set up the standards to confidently distinguish the unfolded and folded status of G-rich regions, for subsequent evaluation of in vivo folding status. To do this, we used two in vitro methods (rG4-seq and SHALiPE-seq in vitro) to provide confident assurance of the correct SHALiPE profiles for in vitro folded RG4s. We then compared the in vivo SHALiPE profiles with the SHALiPE profiles for in vitro folded RG4s. We set up very strict criteria for our comparative in vitro and in vivo data analyses, such as using very high sequencing depth cut-off (average of RT stop reads count ≥ 50 for Gs). We have added a detailed description clarifying how the G-rich regions were selected, highlighted in yellow (line 327 to line 335 on page 16 in our revised manuscript).*

*Following the Referee’s suggestion, we determined RG4 by only using SHALiPE-seq. By directly comparing the Gini difference between in vivo SHALiPE profiles and in vitro Li+ SHALiPE profiles, 831 RG4 sites with Gini in vivo / Gini Li+ ≥ 1.1 were observed (Rebuttal Figure 4A), which are likely to be folded in vivo. Similarly, by directly comparing the Gini difference between in vitro K+ SHALiPE profile and in vitro Li+ SHALiPE profile, we found 819 RG4 sites with (Gini K+/ Gini Li+ ≥ 1.1) which are likely to be folded in vitro (Rebuttal Figure 4B). There is an overlap of 525 RG4 sites between in vitro folded and in vivo folded RG4s (Rebuttal Figure 4B). The large number of overlapped RG4 sites suggests that for most regions that are folded in vitro, they are likely to fold in vivo. Notably, this result is very similar to that using both rG4-seq and SHALiPE-seq, suggesting that using SHALiPE-seq alone is competent for determining the folding status of G-rich regions. As expected, the number of RG4s we determined using both rG4-seq and SHALiPE-seq is certainly smaller (181 regions in Fig. 3b-c) than the number of RG4 determined using SHALiPE-seq only, but provides greater confidence in addressing the existence of RG4s in vivo.*

*Rebuttal Figure 4 RG4 detection using SHALiPE-seq alone on the predicted G-rich regions*

*(A) For the regions with Gini in vivo / Gini Li+ ≥ 1.1, 1,351 and 937 regions were found with average reads count over 50 for SHALiPE-seq in vitro with Li+ or in vivo, respectively, resulting in an overlap of 831 regions.*

*(B) For regions with average reads count over 50 for SHALiPE-seq in vitro with Li+ , in vitro with K+ and in vivo, 819 regions were found with Gini K+ / Gini Li+ ≥ 1.1, while 712 regions were found with Gini in vivo / Gini Li+ ≥ 1.1, resulting in an overlap of 525 regions.*

Comment 3 Related to 1 and 2, is SHALiPE-seq specific for RG4 detection? For Gs that are not in clusters, do you see differences in Gini? How about other nucleotides? This reviewer wonders whether other features of RNA, such as RNA modification or secondary structure, confound the interpretation of SHALiPE-seq for RG4.

*RESPONSE*

*We thank the Referee for this comment. The SHALiPE method is reported to be very specific for RG4 detection from our previous individual in vitro RG4 detection[2]. Here, we extended our SHALiPE method for detecting in vivo RG4s at the genome wide scale. Following the Referee’s suggestion, we calculated the Gini for non-cluster Gs and other non-G nucleotides (Rebuttal Figure 5). When comparing the Gini in vitro with Li+ to that in vitro with K+, we found significant differences only on Gs in clusters (Rebuttal Figure S5A and Fig. 2b) but not on Gs not in clusters, nor on other bases (Rebuttal Figure S5B-C, new Figure S4). Since RG4 was stabilized under K+ conditions, the differences on the Gs in clusters reflect the change of the RG4 folding status. Therefore, SHALiPE-seq is specific for RG4 detection.*

*A previous study revealed that the RNA modification on G is the N7-methylguanosine methylation (m7G). In general, the N7 position of G is not at the Watson-crick face, and thus not able to stall reverse transcriptase. This was also proved by a recent study of m7G in Arabidopsis[6]. Therefore, it is unlikely that m7G RNA modification could confound the interpretation of SHALiPE-seq for RG4. Regarding the query about secondary structures, we found in our study that those regions detected in vitro by rG4-seq are unlikely to fold into strong secondary structures such as stem loop structures (Fig. 1i). In the other words, RG4 tends to fold in the regions with fewer secondary structures, and which cannot cause reverse transcription stalling. Therefore, alongside the specificity of SHALiPE profiles in response to K+, it is unlikely that RNA secondary structures confound the interpretation of SHALiPE-seq.*

*In summary, SHALiPE-seq is specific for RG4 detection.*

*Rebuttal Figure 5 Comparison of Gini values of SHALiPE-seq on folded RG4s in vitro.*

*(A) Violin plot showing Gini in vitro with Li+ and Gini in vitro with K+ on Gs in clusters. Gini with K+ is significantly higher than Gini with Li+ (P < 10-9). Regions with average counts ≥ 50 on the bases were included for calculation. P-value, paired Student’s t-test.*

*(B) Violin plot showing Gini in vitro with Li+ and Gini in vitro with K+ on Gs not in clusters. Gini with K+ is similar to Gini with Li+ (P = 0.92). Otherwise in Rebuttal Figure 5A.*

*(C) Violin plot showing Gini in vitro with Li+ and Gini in vitro with K+ on the other bases (A, C and U). Gini with K+ is similar to Gini with Li+ (P = 0.31). Otherwise in Rebuttal Figure 5A.*

Comment 4 It would be nice to know whether the same genes tend to form RG4s in their RNAs in Arabidopsis and rice. Are there specifically enriched GO terms?

*RESPONSE*

*We thank the Referee for this query. Firstly, we found there are 121 orthologues containing detected RG4s between Arabidopsis and rice (new Table S6), which indicates that these RG4s are likely to be conserved in plants. We have also performed GO analysis using the genes with RG4s in Arabidopsis and rice. Interestingly, the enriched GO items were highly overlapped between Arabidopsis and rice, particularly in the molecular functions related to RNA binding and protein binding (Rebuttal Figure 6, new Figure S6). This further supports our conclusion that folded RG4s are likely to be conserved in plants. We have added descriptions of these results to our revised manuscript in lines from 241 to 247 on page 12, accordingly.*

*Rebuttal Figure* *6 Gene Ontology (GO) analysis reveals enrichment of genes with similar molecular functions in Arabidopsis and rice for genes with RG4s.*

*(A) GO terms of molecular functions enriched for genes with RG4s in Arabidopsis (P < 0.05). P-value, Fisher’s exact-test with Bonferroni correction.*

*(B) GO terms of molecular functions enriched for genes with RG4s in rice (P < 0.05) listed. Otherwise in Rebuttal Figure 6A.*

Comment 5 The authors concluded that G-rich regions that do not form RG4s in vitro are more likely to form secondary structures. This was based on RNA structure prediction. But the authors actually have profiled RNA structures in vivo. Why not confirm their prediction-based conclusion with available information on RNA secondary structures in vivo? They have both in vivo RG4 and in vivo secondary structure information.

*RESPONSE*

*We thank the Referee for this comment.*

*Since our SHALiPE is specifically designed for RG4 detection, not for secondary structure, we used our previous DMS-based in vivo secondary structure data, Structure-seq for comparative analysis[7]. For undetected G-rich regions, the base-pairing probability (BPP) of G-rich regions in in vivo RNA structures were significantly higher compared to flanking regions (Rebuttal Figure 7, P < 10-16, Student’s t-test), indicating these G-rich regions were folded into strong secondary structures in vivo. In contrast, for those detected G-rich regions in the presence of K+, no significant differences of BPPs were found between G-rich regions and flanking sequences (Rebuttal Figure 7, P = 0.116, Student’s t-test), and the BPPs of G-rich regions are much lower than those of undetected G-rich regions (Rebuttal Figure 7, P < 10-16, Student’s t-test). This result agrees well with what we observed using in silico prediction (Fig. 1i).*

*Rebuttal Figure 7 Secondary structure folding represses RG4 formation*

*Comparison of base-pairing probability (BPP) of alternative secondary structures in G-rich regions that are detected purple and undetected (green). RNA structure folding was performed using Vienna RNAfold with DMS reactivity constraints. The Gs in the G-rich region were classed to 8 bins, flanking sequences (100 nt on both sides) were classed to 20 bins, with 15 bins close to G-rich regions shown.*

Comment 6 In addition to the comments above on the bioinformatics analyses, there are concerns on the functional study of the RG4 in HIRD11. Please mention how many independent transgenic lines were evaluated for the rescue experiment. Also, the mutations not only abolish RG4 formation but also potentially change the secondary structure and definitely change the sequence of the RNA, thus the defects associated with the mutant transgene do not necessarily reflect the function of the RG4. It is hard to get around this issue, but at least the authors need to discuss this point.

*RESPONSE*

*We thank the Referee for pointing this out.*

*We have added: “the primary root length of three independent lines of mutRG4 plants was distinctively longer than that of wtRG4 plants”, in lines from 268 to 269 on page 13 of our revised manuscript. “3 representative lines out of 6 independent lines for wtRG4 plants and 9 independent lines for mutRG4 plants were shown” were also added to the Fig. 5f figure legend. We appreciate the Referee’s comment and have also inserted an additional discussion point: “Given the potential effect of sequence change in the mutated RG4s that may affect other post-transcriptional regulations of gene expression, more mutations could be considered in future studies for assessing the biological function of individual RG4s.” in our revised manuscript from line 383 to line 386, on page 19.*

Reviewer #3:

Although G-rich sequences are abundant in eukaryotic cells, little is known the existence of RNA G-quadruplex structure in vivo in plants and how it can control translation and plant development. In this study, Yang et al. determined in vitro (K+, PDS) and in vivo (2-methylnicotinic acid imidazolide (NAI)) RNA chemical structure profiling to determine whether RG4 structures are folded in Arabidopsis and rice. The authors explicitly provided the evidence of RG4 folding formation in vivo via SHALiPE-seq in both plants. They also proved the physiological role of RG4 in vivo by showing that RG4 inhibits translation of HIRD11 gene as an example in Arabidopsis which controls plant root growth. Conclusively, this manuscript illustrates the existence of folded RG4in vivo and emphasizes the functional role of RG4 in plants. However, regardless of the novelty of this study, there are several drawbacks that might hamper to understand the functional roles of RG4s in various
aspects of plant growth and development.

*RESPONSE*

*We thank the Referee for their appreciation that our work: “Conclusively, this manuscript illustrates the existence of folded RG4in vivo and emphasizes the functional role of RG4 in plants”. We appreciate all the insightful suggestions the Referee has made, which have helped us to deliver an improved revision of our manuscript.*

Comment1

Comment1.1 In Figure 1h, it would be informative to make a plot of showing a proportional distribution of RG4s across genes by plotting RG4s in the start-end of all genes with RG4s in 2kb-window. That is, where are RG4s exactly enriched in plant gene structures? Gene ends or the middle of genes or uniform across gene? It might be necessary to compare the sequenced RG4 with predicted in silico RG4.

*RESPONSE*

*We thank the Referee for this comment.*

*Regarding the gene structure in Arabidopsis, most Arabidopsis UTRs are quite short (<500 nt, Rebuttal Figure 8). When filtering the genes with a cut-off of UTRs ≥ 500 nt, and CDS ≥ 1000 nt, only 2 genes passed these cut-off criteria. Following the Referee’s suggestion, we have added predicted counts information (Rebuttal Figure 9A, new Fig. 1h). To help visualize RG4 distribution in an alternative way, we then divided the UTRs into 3 bins and CDS into 10 bins. We found that both sequenced and in silico predicted RG4s are strongly enriched in the CDS region (Rebuttal Figure 9B), which agrees with our conclusion in the manuscript.*

*Rebuttal Figure 8 Length of different genic regions of genes with detected RG4s or predicted G-rich regions in Arabidopsis*

*Histogram showing the length of 5’UTR (A-B), CDS (C-D) and 3’UTR (E-F) of genes with detected RG4s (A, C and E) or predicted G-rich regions (B, D and F), respectively.*

*Rebuttal Figure 9 The prevalence of predicted and detected G-rich regions in different genic regions*

*(A) Bar plot showing the counts of detected RG4s or predicted G-rich regions in 5’UTR, CDS and 3’UTR, respectively, in Arabidopsis.*

*(B) Bar plot showing the bin counts of detected RG4s or predicted G-rich regions in 3 bins for 5’UTR, 10 bins for CDS and 3 bins for 3’UTR, respectively, in Arabidopsis.*

Comment1.2 Why not showing the same graph or plot for in vivo SHALiPE profiles in Figure 3-5? Please consider to add gene structure on rG4seq or SHALiPE seqs (in vivo, in vitro +K+ and Li+) in Figure 1-5, which will show the location of RG4s in gene structure (5´UTR, gene body, 3´UTR).

*RESPONSE*

*We thank the Referee for this comment to help us improve our manuscript.*

*The same regions were used to compare the profiles of different datasets. The two regions showing SHALiPE-seq profiles in Fig. 3d-e were the same two regions used to show rG4-seq profile in Fig.1f-g (AT4G30460 and AT3G23450). We apologize that this point was not clear in our manuscript. We have now clarified this in our revised manuscript (line 219 to line 220 on page 11). The rG4-seq profile on HIRD11 is added in Figure 5b. Gene structures have been added to all the figures as suggested.*

Comment 2. It is shown that RG4s are enriched at CDS in plants. Do these gene body RG4s also affect their corresponding translation? if so, what would be a mechanistic base of gene body-seeded translational regulation? The authors claimed that the location of RG4s in gene doesn't affect RG4 structure folding, but there is no evidence showing how CDS RG4s affect translation in plants, unlike the cases that SMXL5 has RG4 at 5´UTR and HIRD11 at 3´UTR for translation inhibition.

Comment 3. In respect to in vivo functional role of RG4s in translation, it would be necessary to show ribosome-seq profile patterns of wild-type plants at the same plot of RG4 distribution in all genes with RG4s and all genes without RG4s, together with RNA seq profiles if necessary.

*RESPONSE*

*We thank the Referee for these two comments, which we have addressed by performing additional analyses as follows.*

*Previous studies have suggested strong RNA secondary structures in CDS regions are able to repress translation[8]. Therefore, as a strong structure, RG4 in the CDS region is likely to affect translation. Furthermore, we calculated the translation efficiency of transcripts containing either detected RG4s (RG4 detected) or predicted but detected G-rich regions (RG4 undetected) in CDS regions using the ribosome profiling data in Arabidopsis[1]. We found the translation efficiency of those mRNAs containing detected RG4s in the CDS, are significantly lower than the translation efficiency of transcripts with RG4s undetected in CDS regions (Rebuttal Figure 10A). Therefore, this result suggested that RG4s detected in CDS regions generally repress translation. Moreover, we compared the ribo-seq profiles of transcripts with or without RG4s detected in CDS regions, by aligning the start site of the RG4 sites/G-rich regions with the codon reading frames. We found two interesting signatures that may be related to translation. Firstly, the ribo-seq read counts across the start site of the RG4s for RG4 detected transcripts are significantly lower than those for RG4 undetected transcripts (Rebuttal Figure 10B, upper panel, marked by blue stars). This result suggested that fewer ribosomes are occupying RG4 sites. Secondly, there are two peaks of ribo-seq read counts at positions with a distance of 4 and 5 codons upstream of the RG4 start site (Rebuttal Figure 10B, upper panel, marked by orange stars), indicating a high ribosome density at these positions. The high ribosome density may suggest a relatively slow speed and pausing of ribosome elongation[9]. We did not find these two features in the RNA-seq data (Rebuttal Figure 10B, bottom panel). Interestingly, the distance between ribosome pausing peaks and the RG4 start site is around 14nt, similar to the half-length of the occupancy size for one ribosome (Rebuttal Figure 10C). One possible explanation might be that RG4 detected in CDS regions may impede translation elongation (Rebuttal Figure 10C). These preliminary observations provide a new avenue of research for a closer examination of whether these RG4s play a functional role in regulating translation elongation.*

*Rebuttal Figure 10 RNA G-quadruplex structures in CDS regions affect translation*

*(A) Violin plot of translation efficiency (TE) of transcripts with predicted but undetected RG4s (RG4 undetected) or with detected RG4s (RG4 detected) in CDS regions. TE of transcripts with detected RG4s is significantly lower than that of RG4 undetected transcripts (P < 3 × 10-13, Student’s t-test).*

*(B) Line plot showing the profiles of ribo-seq and RNA-seq around G-rich region. The nucleotide of G-rich start site is assigned as 0 position.*

*(C) Diagram showing a hypothetical potential mechanism of RG4 structure in translation elongation. Ribosomes may be paused by the downstream RG4 structure, resulting in higher occupancy of ribosomes on the bases that are about 14nt upstream from the RG4 structures.*

Comment 4. It would be informative if Venn diagrams are presented showing how many genes with RG4s are overlapped and separated between rG4seq or SHALiPE seqs (in vivo, in vitro +K+ and Li+) in Arabidopsis and rice. It would be intriguing if there is any preference of RG4 structures on in vitro, in vivo, or both, which I think give us a hint for prediction and properties of RG4 structure formation.

*RESPONSE*

*We thank the Referee for this comment.*

*Following the Referee’s constructive suggestion, we have added Venn diagrams for Arabidopsis (Rebuttal Figure 3A, new Figure S4) and rice (Rebuttal Figure 11A), respectively. In both Arabidopsis and rice, most folded RG4 sites detected in vitro tend to fold in vivo as well (Rebuttal Figure 3B and Rebuttal Figure 11B). We plotted in detail the numbers of different types of RG4s (Rebuttal Figure 3C and Rebuttal Figure 11C). We found that most of RG4s with different types folded in vitro tend to form in vivo in both Arabidopsis and rice (Rebuttal Figure 3C and Rebuttal Figure 11C). However, there are slightly more G2 RG4s folded in vivo having longer loop lengths (G2L1-4 and G2L1-9), as compared to G2L1-2. These results imply that those G2 RG4 with longer loop lengths may be more strongly folded in vivo, which will be interested in the further investigation.*

*Rebuttal Figure 11 Structural landscape of folded RNA G-quadruplex features in rice*

*(A) Venn diagram showing the number of regions for analysis in each dataset. 187 G-rich regions with average reads count on guanines over 50 were found to overlap in SHALiPE-seq libraries for in vitro conditions with Li+, in vitro with K+ and in vivo respectively. We focused on these 187 regions to calculate in vivo folding scores.*

*(B) In vivo folding score (FS) on the 187 regions in rice, with folding score > 0 on 146 regions while folding score < 0 on the other 41 regions.*

*(C) Counts of regions with folding score > 0 or folding score < 0 of different types of RG4s.*

Comment 5. The authors examined the 5´UTR of SMXL5 to validate rG4seq in Figure 1. Why not showing the representative locus for SHALiPE profiles (in vivo, in vitro +K+ and Li+)? If not, is there any reason?

*RESPONSE*

*We thank the Referee for this comment.*

*SMXL5 is expressed at relatively low levels since it only functions in plant phloem[10]. Although we are able to detect it in our rG4-seq data (counted by sequencing read coverage), it did not exceed our cut-off criterion for SHALiPE-seq libraries (i.e. RT stop reads count ≥ 50 for Gs). We attach both rG4-seq and SHALiPE-seq profiles for the 5´UTR of SMXL5 (Rebuttal Figure 12). In the future it will be interesting to pursue the SHALiPE probing of RG4 on the 5´UTR of SMXL5 by only enriching the RNAs in phloem.*

*Rebuttal Figure 12 RG4-seq and SHALiPE-seq profiles of AtSMXL5*

*(A-C) rG4-seq profiles of full-length transcript of AtSMXL5.*

*(D-F) SHALiPE-seq profiles of full-length transcript of AtSMXL5.*

*(G-I) Expanded rG4-seq profiles of AtSMXL5 fragment from 220-350 nt. The red line indicates the G-rich region located in 5’UTR of AtSMXL5. The translation start codon AUG is indicated with black triangle.*

*(J-L) Expanded SHALiPE-seq profiles of AtSMXL5 fragment from 220-350 nt.*

Comment 6. In Figure 5 and Fig S4, mutated RG4 HIRD11 transcripts were shown to be enriched in polysome fraction and no change in mRNA level. Is there any change in protein level?

*RESPONSE*

*We thank the Referee for this comment.*

*Since there is no commercial antibody against HIRD11, we used polysome analysis to evaluate the translation level of HIRD11. The polysome analysis by combining sucrose gradient fragmentation with qRT-PCR is commonly used in assessing the translation level of genes in plant research field due to a lack of commercial antibody for most plant genes [11, 12]. To further confirm the translation suppression by this RG4, we performed dual luciferase reporting assay to assess the impact of RG4 on protein levels. We found that the Firefly luciferase activity (F-LUC) of mutRG4 was ~3 times higher to that of wtRG4, when normalized to Renilla luciferase (R-LUC) activity used as the internal control (Rebuttal Figure 1A and new Figure S8, P < 10 -11, Student’s t-test), while RNA abundance is similar (Rebuttal Figure 1B). Therefore, the RG4 on HIRD11 is able to repress translation. These new results are described from line 278 to line 284 on page 13 in the revised manuscript.*

Comment 7. Given that functional RG4 structures, which are known to be globally unfolded in mammalian and yeast cells, exist in Arabidopsis and rice, I wonder whether RG4 structures are conserved in plants. To this end, it might be helpful to analyze sequence homology of genes with RG4 structure in Arabidopsis and rice.

*RESPONSE*

*We thank the Referee for this comment.*

*Among the RG4s we detected in both Arabidopsis and rice, we found 121 orthologue pairs between Arabidopsis and rice, containing conserved RG4 sites (new Table S6). For instance, the orthologue pair of AT3G62790 and LOC_Os08g44250 contain conserved RG4 sites in the CDS region (Rebuttal Figure 13). The orthologue pair of AT2G16500 and LOC_Os06g04070 contain conserved RG4 sites in the 5’UTR region (Rebuttal Figure 13). We have added the additional data of these 121 homologue pairs in the new Table S6.*

*Rebuttal Figure 13 Conserved RG4 regions on orthologue pairs of Arabidopsis and rice*

*Two examples are shown, on the pairs of AT3G62790 and LOC_Os08g44250, AT2G16500 and LOC_Os06g04070. The locations of the RG4 regions on these genes are indicated by orange squares.*

Minor comments;

1. In page 10 line 14, 'presence of K+ (0.67 and 0.52)' should be 'presence of K+ (0.67 and 0.56)'.

*RESPONSE*

*We thank the Referee for pointing this out and have corrected the number, highlighted in yellow (line 192 on page 10), accordingly.*

2. In the method part for alignment of the sequencing reads, it would be helpful to provide parameters for bowtie alignment and handling process of aligned read such as filtering of mapping quality, how multiple mapped reads were processed.

*RESPONSE*

*We thank the Referee for this comment. The details of parameters for bowtie alignment and handling process of aligned reads followed standards from our previous detailed protocols[7, 13]. We have added this information in our revised manuscript (from line 518 to line 520 on page 26).*

3. In Gini index, page 26 line 27 for 'G residues in the G-tracts', what does the G-tracts present? Dose it mean G-rich regions? How G-tracts were defined?

*RESPONSE*

*We thank the Referee for this comment and have added the definition of G-tracts “continuous runs of guanine in the G-rich region” in our revised manuscript, highlighted in yellow (line 559 to line 560 on page 28), accordingly.*

4. In Gini index, what γ¯ does stand for the mean of reads number of γi ?

*RESPONSE*

*We thank the Referee for pointing out this point. We have now added further explanation: “and* $\overline{r}$ *denotes average reads number of all G residues”, from line 560 to line 561 on page 28, accordingly.*

*References*

*1.	Liu MJ, Wu SH, Wu JF, Lin WD, Wu YC, Tsai TY, Tsai HL, Wu SH: Translational landscape of photomorphogenic Arabidopsis. Plant Cell 2013, 25:3699-3710.*

*2.	Kwok CK, Sahakyan AB, Balasubramanian S: Structural Analysis using SHALiPE to Reveal RNA G-Quadruplex Formation in Human Precursor MicroRNA. Angew Chem Int Ed Engl 2016, 55:8958-8961.*

*3.	Kwok CK, Marsico G, Balasubramanian S: Detecting RNA G-Quadruplexes (rG4s) in the Transcriptome. Cold Spring Harb Perspect Biol 2018, 10.*

*4.	Wang Z, Wang M, Wang T, Zhang Y, Zhang X: Genome-wide probing RNA structure with the modified DMS-MaPseq in Arabidopsis. Methods 2019, 155:30-40.*

*5.	Pandey S, Agarwala P, Jayaraj GG, Gargallo R, Maiti S: The RNA Stem-Loop to G-Quadruplex Equilibrium Controls Mature MicroRNA Production inside the Cell. Biochemistry 2015, 54:7067-7078.*

*6.	Enroth C, Poulsen LD, Iversen S, Kirpekar F, Albrechtsen A, Vinther J: Detection of internal N7-methylguanosine (m7G) RNA modifications by mutational profiling sequencing. Nucleic Acids Res 2019, 47:e126.*

*7.	Norris M, Kwok CK, Cheema J, Hartley M, Morris RJ, Aviran S, Ding Y: FoldAtlas: a repository for genome-wide RNA structure probing data. Bioinformatics 2017, 33:306-308.*

*8.	Mustoe AM, Busan S, Rice GM, Hajdin CE, Peterson BK, Ruda VM, Kubica N, Nutiu R, Baryza JL, Weeks KM: Pervasive Regulatory Functions of mRNA Structure Revealed by High-Resolution SHAPE Probing. Cell 2018, 173:181-195 e118.*

*9.	Dana A, Tuller T: Determinants of translation elongation speed and ribosomal profiling biases in mouse embryonic stem cells. PLoS Comput Biol 2012, 8:e1002755.*

*10.	Cho H, Cho HS, Nam H, Jo H, Yoon J, Park C, Dang TVT, Kim E, Jeong J, Park S, et al: Translational control of phloem development by RNA G-quadruplex-JULGI determines plant sink strength. Nat Plants 2018, 4:376-390.*

*11.	Missra A, von Arnim AG: Analysis of mRNA translation states in Arabidopsis over the diurnal cycle by polysome microarray. Methods Mol Biol 2014, 1158:157-174.*

*12.	Li W, Ma M, Feng Y, Li H, Wang Y, Ma Y, Li M, An F, Guo H: EIN2-directed translational regulation of ethylene signaling in Arabidopsis. Cell 2015, 163:670-683.*

*13.	Ding Y, Kwok CK, Tang Y, Bevilacqua PC, Assmann SM: Genome-wide profiling of in vivo RNA structure at single-nucleotide resolution using structure-seq. Nat Protoc 2015, 10:1050-1066.*

**Second round of review**

**Reviewer 1**

The authors have fully addressed the concern that I have raised and I am supportive of publication. I would like to thank the authors for their efforts in addressing experimentally my comments despite limitations to lab access due to the COVID-19 pandemic, I believe the addition of these experiments have significantly strengthen the manuscript.

**Reviewer 2**

The authors have fully addressed my questions/comments.

**Reviewer 3**

In the revised manuscript, the authors well addressed the major concerns and questions raised in the original submission.
